# Quantitative investigation of pengornithid enantiornithine diet reveals macrocarnivorous ecology evolved in birds by Early Cretaceous

**DOI:** 10.1016/j.isci.2023.106211

**Published:** 2023-02-16

**Authors:** Case Vincent Miller, Michael Pittman, Xiaoli Wang, Xiaoting Zheng, Jen A. Bright

**Affiliations:** 1 Department of Earth Sciences, the University of Hong Kong, Pokfulam, Hong Kong SAR, China; 2 School of Life Sciences, the Chinese University of Hong Kong, Shatin, Hong Kong SAR, China; 3 Institute of Geology and Paleontology, Linyi University, Linyi, Shandong 276005, China; 4 Shandong Tianyu Museum of Nature, Pingyi, Shandong 273300, China; 5 Department of Biological and Marine Sciences, University of Hull, Hull HU6 7RX, UK

**Keywords:** Ornithology, Evolutionary biology, Paleobiology

## Abstract

The diet of Mesozoic birds is poorly known, limiting evolutionary understanding of birds’ roles in modern ecosystems. Pengornithidae is one of the best understood families of Mesozoic birds, hypothesized to eat insects or only small amounts of meat. We investigate these hypotheses with four lines of evidence: estimated body mass, claw traditional morphometrics, jaw mechanical advantage, and jaw finite element analysis. Owing to limited data, the diets of *Eopengornis* and *Chiappeavis* remain obscure. *Pengornis*, *Parapengornis*, and *Yuanchuavis* show adaptations for vertebrate carnivory. *Pengornis* also has talons similar to living raptorial birds like caracaras that capture and kill large prey, which represents the earliest known adaptation for macrocarnivory in a bird. This supports the appearance of this ecology ∼35 million years earlier than previously thought. These findings greatly increase the niche breadth known for Early Cretaceous birds, and shift the prevailing view that Mesozoic birds mainly occupied low trophic levels.

## Highlights

• Pengornithidae is a well known family of Early Cretaceous toothed birds
• Past hypotheses of their diet indicates that they ate invertebrates or only small amounts of meat
• Combining four lines of evidence, we find several pengornithids most likely ate fish
• *Pengornis* likely ate large animal prey, the earliest evidence of this in a bird

## Graphical abstract

**Figure:**
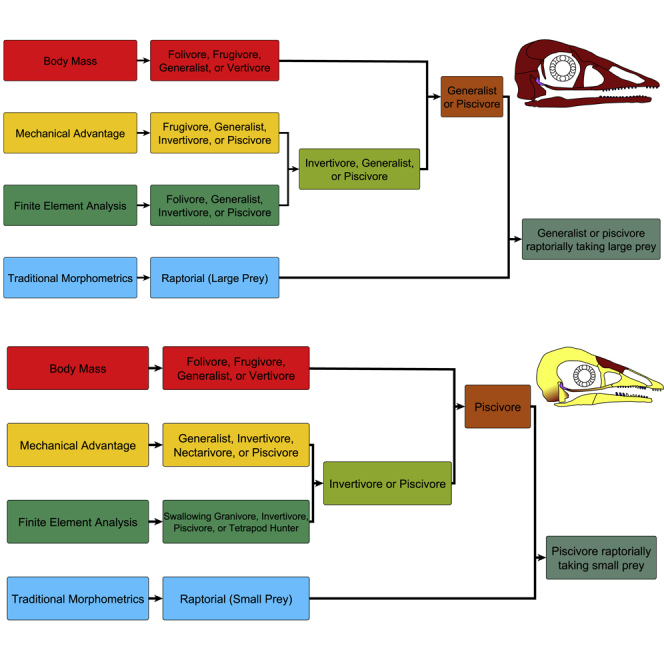

## Introduction

Birds play vital roles in modern ecosystems that are well studied and understood based on a range of evidence including their dietary ecology.^1^ However, how and when birds came to play such important ecological roles remains obscure. Enantiornithine birds dominated the Mesozoic world in both species diversity and geographic range,^2^ and are commonly regarded as the Mesozoic’s ecological equivalent to crown birds.^3^ Thus, understanding enantiornithine ecology is paramount to understanding the origin of the vital roles played by birds in modern ecosystems. Although recent studies of early birds, including enantiornithines, have made great strides in understanding their growth,^4^ reproduction,^5^ and locomotion,^6^ their diet remains largely unknown and a major barrier to fully understanding their ecological roles.

To start to close this large knowledge gap, a recent study investigated the diet of the enantiornithine family Longipterygidae^7^ within a framework of four quantitative lines of evidence: body mass estimation, traditional morphometrics, mechanical advantage and functional indices, and finite element analysis.^8^ This study supported the prevailing view that early birds occupied low trophic levels.^9^^,^^10^^,^^11^

To evaluate this ‘low trophic level’ (LTL) hypothesis further, this study investigates the diet of pengornithids. Pengornithids are among the most early diverging enantiornithine lineages, appearing by at least the Hauterivian stage of the Early Cretaceous.^2^ The family currently has five recognized members: *Chiappeavis*,^12^^,^^13^ *Eopengornis*,^14^ *Parapengornis*,^15^ *Pengornis*,^16^ and *Yuanchuavis*.^17^ Pengornithids have been previously hypothesized to inhabit low trophic levels. The round, low crowned teeth of *Pengornis* have been interpreted as adaptations for consumption of invertebrates^18^^,^^19^ pg. 136 or hard-bodied prey^20^ pg. 83. Those of other pengornithids are more conical but still low-crowned, which has been interpreted by^9^ pg. 191 as evidence of hypocarnivory (eating little meat).

However, pengornithids also have other features that imply a different ecology. Pengornithids are larger than most Early Cretaceous enantiornithines^8^^,^^21^ and are exceeded only by the Late Cretaceous avisaurids and *Elsornis*.^8^^,^^22^ This trait has the potential to affect the reconstructed diet of pengornithids, because diet is closely linked to body size in modern birds.^23^^,^^24^ Pengornithids also have the largest enantiornithine tail fans in terms of feather count (8–10), which has been interpreted as improving flight performance^12^^,^^17^ and in turn could allow for a more active predatory lifestyle. Together with the fact that pengornithids represent some of the best preserved enantiornithine fossils,^18^ Pengornithidae is an ideal study subject for evaluating the LTL hypothesis.

We improve the framework of^7^ by expanding the extant dataset, reworking ecological categories, and using more appropriate analytical methods. Twenty new extant bird taxa were incorporated to increase representation of dietary categories (Table 1) that previously had small sample sizes (frugivores, granivores, and nectarivores), and to increase the phylogenetic breadth of the sample (ten new families and two new orders are included for the first time). The resulting dataset broadly samples birds phylogenetically and ecologically including ratites, galloanserines, penguins, flamingos, mousebirds, raptors, and parrots among others. We also investigated some of the hypotheses proposed by^7^ that certain ecological categories needed to be split or merged, and overhauled the categories accordingly. This showed that subcategories of invertivores and frugivores proved to be indistinct (see supplemental information), and led to changes to the categorization of raptorial styles originally used by.^7^^,^^25^^,^^26^ In this study they have been simplified to two categories which previous work on raptors^27^ found to be meaningful and which we find to better explain the data: (1) Raptors which take small prey, i.e., prey that can be completely encircled by their foot; and (2) raptors that take large prey, i.e. prey that cannot be completely encircled by their foot.

**Table 1:** Cut-offs for diets used in this study

Diet	Cut-Off
Folivore	60+% Diet-PlantO
Frugivore	60+% Diet-Fruit
Generalist	40% or less in any category
Granivore	70+% Diet-Seed
Invertivore	60+% Diet-Inv
Nectarivore	60+% Diet-Nect
Piscivore	50+% Diet-Fish
Scavenger	50+% Diet-Scav
Tetrapod Hunter	60+% Diet-Tetr

Percentages refer to values given in EltonTraits 1.0, with Diet-Tetr being the sum of Diet-Ect and Diet-End (i.e., ectothermic and endothermic tetrapods are combined).

Here we use our data to test the low trophic level hypothesis using pengornithids. All four lines of evidence are synthesized when interpreting diet, allowing for a more precise and robustly supported diet assignment than any individual line of evidence.^8^ We ultimately find hypotheses of invertivory and hypocarnivory to be unlikely in pengornithids, and *Pengornis* to display the earliest evidence of macrocarnivory among avialans. This would place it at a high trophic level, quantitatively refuting the LTL hypothesis for the first time in an early bird.

## Results

### Body mass

Pengornithid body masses have been estimated previously^8^^,^^21^ and are provided in Table S1 alongside one new mass calculation. The extant avian body mass dataset of our previous work^7^ was expanded with 20 new taxa (new n = 141) as well as updates to diet categories (see STAR Methods). This revised extant body mass dataset was investigated by diet category to identify trends and compare to pengornithid body masses.

**Figure 1:**
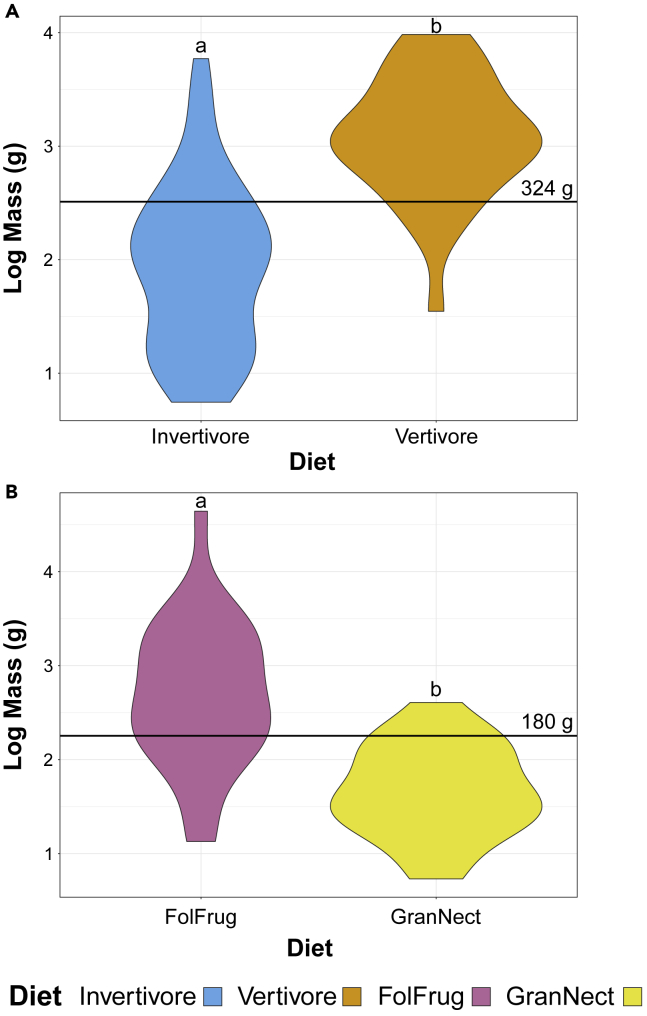
Violin plots of extant bird mass, lumped based on trends apparent in Figure S1B Diets with the same letter are not significantly different in phylogenetic HSD at the p = 0.05 level (Table S2). Cut-off points, calculated using the Youden index (see STAR Methods), are labeled with a line. (A) Carnivores split into invertivores and vertivores. (B) Herbivores split into Folivores + Frugivores (FolFrug) and Granivores + Nectarivores (GranNect).

Violin plots of body masses in extant birds organized by diet are provided in Figures 1 and S1. Table S2 provides pvalues testing if diet means are significantly different with Tukey’s HSD^28^ and phylogenetic HSD.^29^ Carnivore, herbivore, and omnivore masses are not significantly different from one another. Masses of vertivores are significantly different from invertivores in Tukey’s and phylogenetic HSD, as are folivores + frugivores and granivores + nectarivores. Optimizing the Youden Index (a summary measure commonly used to select cut-off points in medicinal diagnostics^30^), the optimal cut-off point between these groups are 324 g and 180 g, respectively (Figure 1).

Statistically significant phylogenetic signal is present in all mass datasets of extant birds (Table S3). K is a univariate statistic measuring the phylogenetic signal relative to a Brownian motion model.^31^ K is 1.77 across diets, 1.59 when only considering carnivores, and 1.95 when only considering herbivores.

### Traditional morphometrics

Extant TM data is unchanged from our previous work,^7^ with only pengornithid data added. Ecological categories for the data have been modified to better reflect trends observed in,^7^ see STAR Methods for explanation.

**Figure 2:**
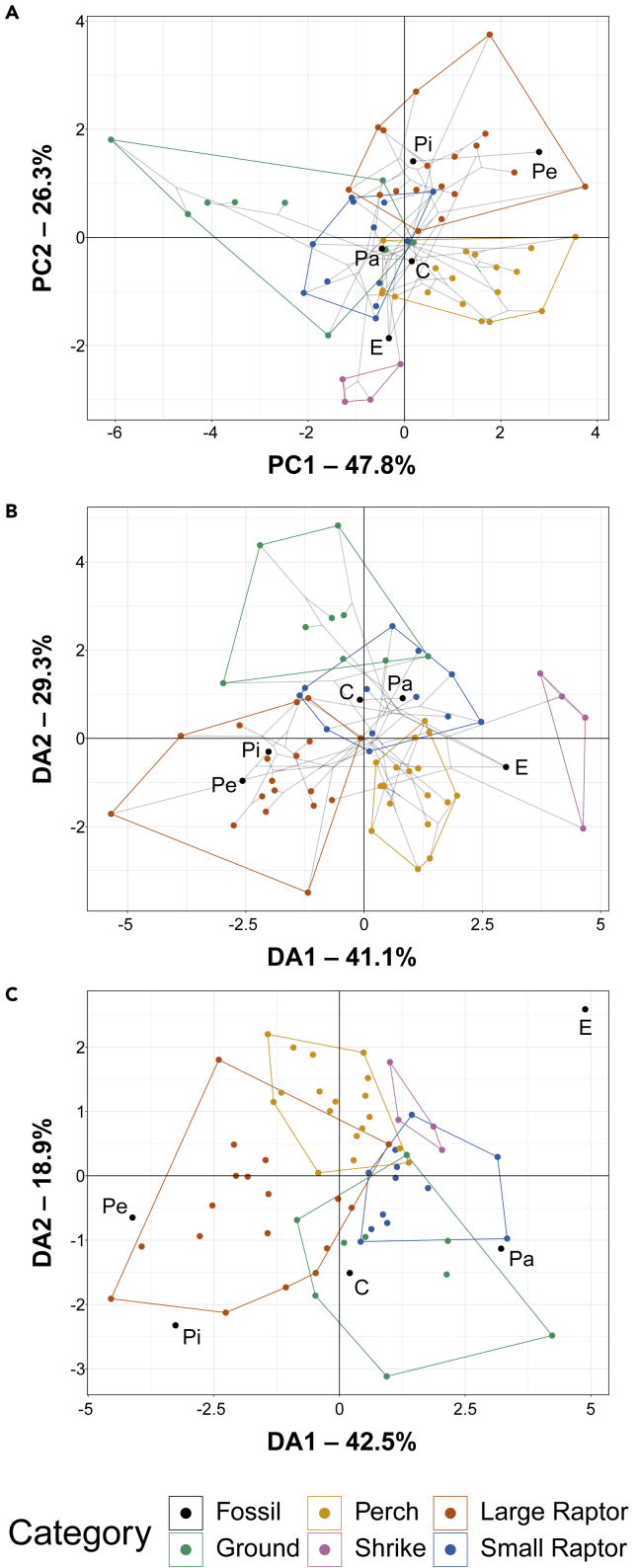
Phylomorphospace of extant avian unguals and pengornithid unguals, based on traditional morphometrics, grouped by pedal ecology Gray lines indicate phylogenetic relationships. Data are visualized with PCA (A), FDA (B), and pFDA (C). In PCA (A), PC1 describes talon curvature and PC2 describes interdigital size variation. In FDA (B), DA1 describes the size ratio of digits II and IV to digit III and DA2 describes the size ratio of digits I and II to digit III. In pFDA (C), DA1 and DA2 are primarily driven by the size ratios of DII and DIV to DIII. See Figure S2 for precise character weights. Taxon abbreviations: C, *Chiappeavis*; E, *Eopengornis*; Pa, *Parapengornis*; Pe, *Pengornis*; Pi, Pengornithidae indet. IVPP V18632.

A principal component analysis (PCA) plot of TM data is provided in Figure 2A with character weights plotted in Figure S2A. An interactive 3D graph is available in the Mendeley data repository. Both *Pengornis* and the indeterminate pengornithid IVPP V18632 plot among large raptors. *Chiappeavis* plots among non-raptorial perching birds. *Eopengornis* plots in an unoccupied region near shrikes and small raptors. *Parapengornis* plots in an indistinct region occupied by ground birds, small raptors, and non-raptorial perching birds. *Parapengornis* is more distinct from non-raptorial perching birds along PC3.

A flexible discriminant analysis (FDA) plot of TM data is provided in Figure 2B with character weights plotted in Figure S2B. An interactive 3D graph is available in the Mendeley data repository. *Pengornis* and the indeterminate pengornithid IVPP V18632 plot within the large raptor space. *Parapengornis* and *Chiappeavis* plot in a region inhabited exclusively by small raptors. *Eopengornis* plots in an unoccupied region between small raptors, shrikes, and non-raptorial perching birds. Discriminant predictions (Table S4) find *Pengornis* and the indeterminate pengornithid IVPP V18632 most likely to have been large raptors, *Parapengornis* as most likely to be a small raptor, and *Eopengornis* most likely to exhibit shrike-like behavior. *Chiappeavis* is recovered as most likely to be a ground bird, but nearly as likely to have any pedal ecology other than shrike-like.

A phylogenetic flexible discriminant analysis (pFDA)^32^ plot of TM data is provided in Figure 2C with character weights plotted in Figure S2C. An interactive 3D graph is available in the Mendeley data repository. Most pengornithids plot outside the extant morphospace, but *Chiappeavis* plots among ground birds. *Pengornis* and the indeterminate pengornithid IVPP V18632 plot adjacent to the large raptor space. *Parapengornis* plots adjacent to the morphospace of ground birds and small raptors. *Eopengornis* plots far outside the region of all extant birds, but closest to shrikes and small raptors. Discriminant predictions (Table S4) find *Pengornis* and IVPP V18632 as most likely to be small raptors, *Parapengornis* and *Eopengornis* most likely to exhibit shrike-like behavior, and *Chiappeavis* most likely to be a non-raptorial perching bird.

Phylogenetic HSD results comparing extant ecological categories are given in Table S5. Ground birds are significantly different from all groups except small raptors at the p < 0.05 level (p = 0.054 ground bird versus small raptor). Small and large raptors are also significantly different at the p < 0.05 level. K_mult_ and K values are unchanged from^7^ (Tables S3 and S6).

### Mechanical advantage

#### Univariate

All functional indices from our previous work^7^ were collected from the upper and lower jaw of each extant bird in that study as well as 20 additional extant birds and pengornithids. Diet categories have also been updated, see STAR Methods for explanation.

Univariate comparisons of functional indices (Figures S3 and S4) show little that is diagnostic between diets. Groups broadly overlap, though some diets have uniquely high or low values of certain indices. Folivores have a high jaw-opening mechanical advantage (OMA) in the upper jaw (Figure S3E). Husking granivores have high anterior and posterior jaw-closing mechanical advantage (AMA and PMA) in the upper jaw (Figures S3A and S3C) and high relative maximum mandibular height (MMH; Figure S4D). Invertivores have a low relative average mandibular height (AMH; Figure S4F). Piscivores have a low relative articular offset (AO) in the lower jaw (Figure S4B), low relative average height of the cranium (ACH; Figure S4E), low MMH (Figure S4D), and low AMH (Figure S4F). Pengornithids have a low AO in the lower jaw (Figure S4B).

#### Multivariate

**Figure 3:**
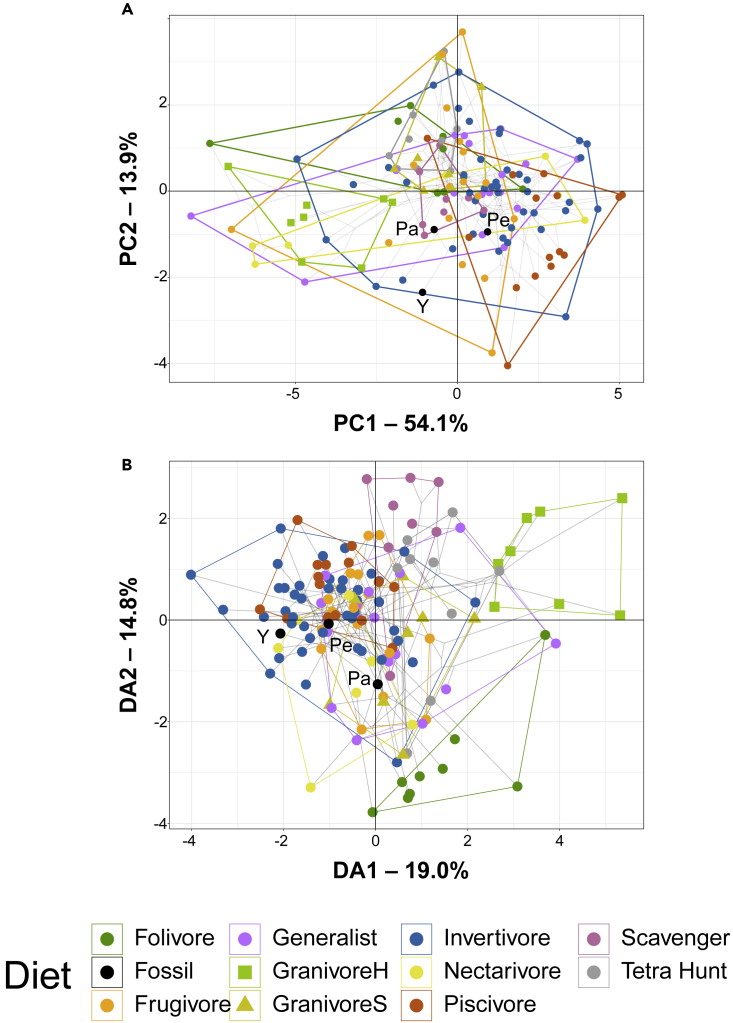
Functional phylomorphospace of extant avian jaws and pengornithid jaws, based on mechanical advantage and functional indices, grouped by diet Gray lines indicate phylogenetic relationships. Data are visualized with PCA (A) and FDA (B). In PCA (A), PC1 is driven primarily by ACH, AMA of both jaws, AMH, and MMH (all in the negative direction). PC2 is driven primarily by lower jaw AO in the positive direction and PMA of both jaws in the negative direction. In FDA (B), DA1 is driven by primarily by upper jaw AMA, upper jaw OMA, and PMA and DA2 is primarily driven by lower jaw AMA, PMA, and OMA, upper jaw AO, MMH, AMH, and ACH. See Figure S5 for precise character weights. Diet abbreviations: GranivoreH, husking granivore; GranivoreS, swallowing granivore; Tetra Hunt, tetrapod hunter. Taxon abbreviations: Pa, *Parapengornis*; Pe, *Pengornis*; Y, *Yuanchuavis*.

PCA plots of MA and functional index data are provided in Figure 3A with character weights plotted in Figure S5A. An interactive 3D graph is available in the Mendeley data repository. *Pengornis* and *Parapengornis* plot in a region occupied by all diets but husking granivores. *Yuanchuavis* plots near invertivores, frugivores, generalists, and piscivores.

FDA plots of MA and functional index data are provided in Figure 3B, with character weights plotted in Figure S5B. An interactive 3D graph is available in the Mendeley data repository. Pengornithids other than *Yuanchuavis* plot in the region of heavy diet overlap, though all but *Pengornis* plot outside the piscivore space. *Yuanchuavis* plots in a region only populated by invertivores. Discriminant predictions (Table S7) find all pengornithids likely to be invertivores, generalists, or piscivores, and unlikely to be husking granivores or tetrapod hunters. *Yuanchuavis* is also recovered as likely to be a nectarivore.

Phylogenetic HSD results comparing MA and functional indices for extant diet categories are given in Table S8. Skull mechanics of piscivores are significantly different from folivores, frugivores, generalists, husking granivores, and invertivores at the p ≤ 0.001 level; from swallowing granivores at the p < 0.01 level; and from tetrapod hunters at the p < 0.05 level. Husking granivores are significantly different from generalists, invertivores, nectarivores, and scavengers at the p < 0.01 level; and folivores and tetrapod hunters at the p < 0.05 level. Scavengers are significantly different from generalists at the p < 0.01 level and folivores at the p < 0.05 level.

Statistically significant phylogenetic signal is present in MA and functional index data overall (Table S3), and in each individual input variable (Table S9). K_mult_ is 0.75 (Table S3) for MA and functional index data. K values for individual MA and functional index measurements (Table S9) range from 0.49 to 1.23, with all but ACH below 1. On average K values are similar for measures of the upper and lower jaws ($\bar{x}$ = 0.79 upper, 0.77 lower), though the upper jaw has a greater range of K than the lower jaw (K = 0.48–1.23 upper jaw, K = 0.61–0.90 lower jaw).

### Finite element analysis

#### Univariate

FEA data follows our previous work^7^ with the addition of 20 new extant taxa and pengornithids. Diet categories have also been updated, see STAR Methods for explanation.

Mesh-weighted arithmetic mean (MWAM) strain^33^ in the lower jaw is plotted by diet in Figure S6. MWAM strain ranges from 57 to 439 με, with an average of 194 με. Most diets overlap in strain ranges. Husking and swallowing granivore MWAM strains overlap minimally, with an apparent transition from swallowing to husking once values exceed 150 με. High MWAM strains (με >275) are mostly restricted to invertivores and piscivores, though one nectarivore (*Promerops cafer*, the Cape sugarbird) also reaches high MWAM strain (397 με). The model of *Pengornis* experiences an MWAM strain of exactly 275 με, all other pengornithid models experience less strain.

#### Multivariate

For datasets based on the intervals method of interpreting finite element models,^34^ PCA results converge at 35 intervals and FDA results converge at 90 intervals.

**Figure 4:**
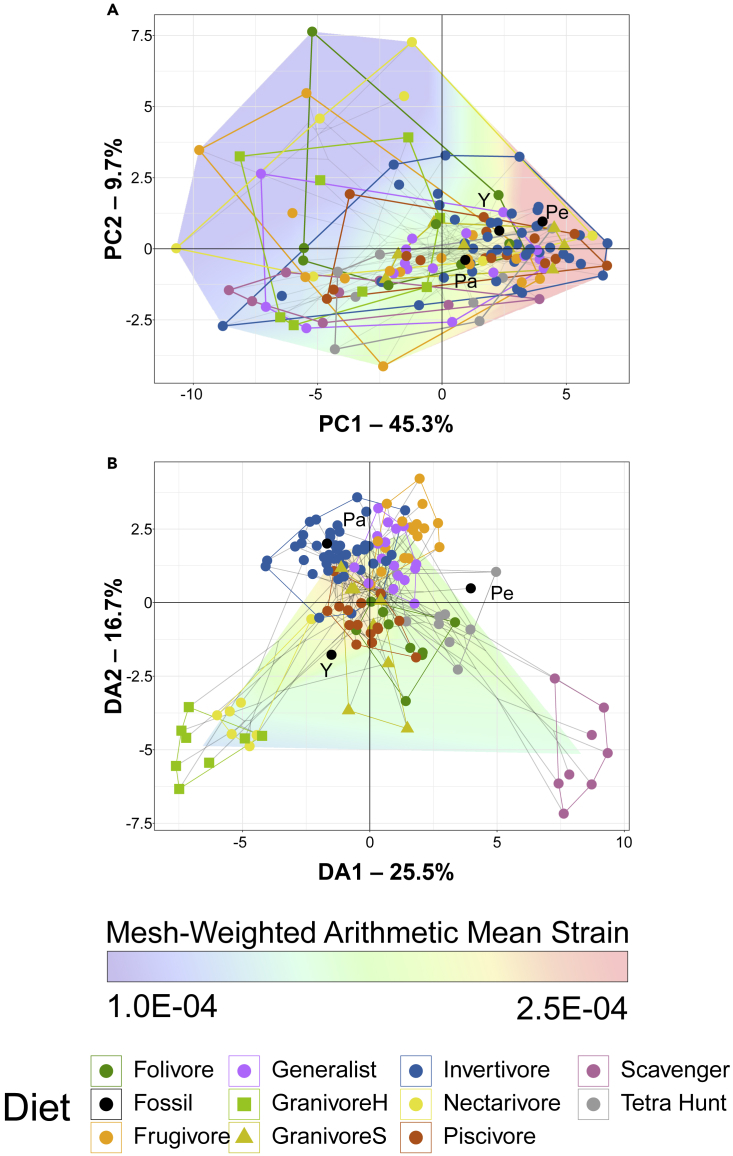
Phylogenetic strain-space of total maximum in-plane principal strain of extant and fossil bird lower jaw finite element models in this study Gray lines indicate phylogenetic relationships. Data are visualized with PCA (A) and FDA (B). Results are obtained using the intervals method^34^ where the percentage of model area under intervals of strain are treated as variables for multivariate analysis. 35 intervals were used for PCA and 90 intervals were used for FDA. In PCA (A), overall strain increases along PC1 and unevenness of strain distribution increases along PC2. In FDA (B), DA1 and DA2 have loadings made of various low-strain intervals, with high-strain intervals clustering near the origin. See Figure S7 for precise character loadings. Diet abbreviations: GranivoreH, husking granivore; GranivoreS, swallowing granivore; Tetra Hunt, tetrapod hunter. Fossil taxon abbreviations: Pa, *Parapengornis*; Pe, *Pengornis*; Y, *Yuanchuavis*.

PCA plots of FEA intervals data (‘strain-space’) are provided in Figure 4A with character weights plotted in Figure S7A. An interactive 3D graph and an interactive graph with points annotated with their contour plots is available in the Mendeley data repository. *Pengornis* plots at positive PC1 and PC2 (weakest jaw area, with slightly more concentrated strain) of the strain-space. *Yuanchuavis* and *Parapengornis* plot in areas of more negative PC1 and PC2, intermediate in value of total strain and strain concentration.

FDA plots of FEA intervals data are provided in Figure 4B with character weights plotted in Figure S7B. An interactive 3D graph is available and a graph with interactive contour plots is available in the Mendeley data repository. Pengornithid jaws spread across the strain-space. *Parapengornis* plots among invertivores. *Pengornis* plots within the tetrapod hunter convex hull but far from the main cluster. *Yuanchuavis* plots in an unoccupied region nearest swallowing granivores and piscivores. Discriminant predictions (Table S10) find invertivory and piscivory somewhat likely for all pengornithids, but neither is the most likely prediction for any taxon. Swallowing granivory is recovered as most likely for *Parapengornis* and *Yuanchuavis*, and generalist feeding is most likely for *Pengornis* (also likely for *Yuanchuavis*). *Pengornis* is unique among pengornithids in also having some affinity with folivores.

Phylogenetic HSD results comparing strain intervals of extant diet categories are given in Table S11. Scavengers are significantly different from all other diets: from generalists and invertivores at the p ≤ 0.001 level; from frugivores, husking and swallowing granivores, and tetrapod hunters at the p < 0.01 level; and from folivores, nectarivores, and piscivores at the p < 0.05 level. Invertivores are significantly different from folivores, generalists, and husking granivores at the p < 0.05 level. Folivores are significantly different from husking granivores at the p < 0.05 level. These differences are noted above the violin plots in Figure S6.

No statistically significant phylogenetic signal was detected in the intervals data (Table S3). The returned K_mult_ value is 0.33. Because of this, pFDA is not appropriate to apply to the FEA intervals data.

## Discussion

### Body mass

Body mass is phylogenetically conserved in extant birds, and herbivorous diets separate more from one another with the changes to the extant dataset (see the supplemental discussion for details).

Predicted body masses for pengornithids range from 155 g as a lower estimate for *Eopengornis* and 556 g as an upper estimate for *Chiappeavis*^8^ (Table S1). *Pengornis*, *Parapengornis*, and *Chiappeavis* have lower body mass estimates above both mass cut-off points (see results), making them more likely to be folivores, frugivores, or vertivores. *Yuanchuavis* is similar in subjective size to these taxa, so the same diets are tentatively proposed. The mass range of *Eopengornis* contains the cut-off point for herbivores and falls below that for carnivores, but because of the incomplete growth of the specimen this line of evidence is considered inconclusive to be conservative. *Chiappeavis* was likely unusually large among pengornithids. The only known specimen of *Chiappeavis* is the most immature described pengornithid,^13^ so a fully mature individual is expected to be significantly larger than other pengornithids.

### Traditional morphometrics

Changes to pedal ecological categories herein create more distinct separation in the extant TM data and further support an ecological driver of talon shape over a phylogenetic one (see the supplemental discussion for details).

Pengornithid claws have a range of curvature and interdigital size variation, but all but *Chiappeavis* are most similar to birds which use their feet in taking prey. Both *Pengornis* and the indeterminate pengornithid IVPP V18632 plot among large raptors in PCA, FDA, and pFDA (Figure 2), and both are predicted to be large raptors with greater than 90% confidence by FDA (Table S4). pFDA predicts *Pengornis* to be a small raptor with over 90% confidence (Table S4). However, as *Pengornis* plots closer to large raptors than small raptors along every pFDA axis, the reason for this posterior pFDA prediction of *Pengornis* as a small raptor is unclear. This result is therefore considered with some caution. Subjectively, its toe joints are also strongly hinged (=“ginglymoid” *sensu*^26^) (Figure 2G in^14^), as expected in a pes adapted for grasping.^26^ Thus, the foot of *Pengornis* is most similar to those of extant large raptors. *Parapengornis* plots among small raptors in FDA (Figure 2B) and is predicted by FDA as a small raptor with over 90% confidence (Table S4). In pFDA it plots outside extant birds closest to small raptors and shrikes (Figure 2C), with predictions confidently placing it in the shrike category (Table S4). *Parapengornis* has a fourth toe longer than its second, which is considered a grasping adaptation.^26^ It also has weakly hinged toe articulations, indicating some grasping adaptation^26^ but less than *Pengornis*. Thus, *Parapengornis* is considered equally likely to have been a small raptor or shrike-like. The hypothesis that *Parapengornis* was scansorially specialised^15^ cannot be directly tested because of this dataset not including any climbing specialists, though *Parapengornis* plots in a region of intermediate claw curvature (Figure 2A) whereas the claws of woodpeckers (Picidae)^35^ and tree creepers (Certhiidae)^36^ are both reported as highly recurved. *Eopengornis* plots in regions outside of any extant group’s convex hull, though it is always closest to shrikes (Figure 2). Discriminant predictions also consistently find it most likely to be shrike-like (Table S4). *Eopengornis*’ fourth toe is much longer than its second,^14^ and its toe joints are somewhat hinged (between *Pengornis* and *Parapengornis*) pointing to grasping adaptations intermediate between *Pengornis* and *Parapengornis*. *Eopengornis* is interpreted as most likely having used its pes in a shrike-like manner: some use in restraining prey, but only for short periods and not usable as a method of killing. *Chiappeavis* occupies a region exclusive to non-raptorial perching birds in PCA, to small raptors in FDA, and to ground birds in pFDA. Discriminant predictions from FDA find its claws most similar to those of ground birds, whereas those of pFDA assign it to non-raptorial perching birds. Its phalanges generally appear weakly hinged, not well-suited for grasping, but their eroded nature makes this uncertain (CVM and XW pers. obs.). Although *Chiappeavis* cannot be confidently assigned to a specific pedal ecology, it does not show adaptations for taking prey with its talons. The early ontogenetic stage of the only known specimen of *Chiappeavis* would not be expected to affect this result, as^25^^,^^26^ demonstrated that an ontogenetic series of great horned owl (*Bubo virginianus*) talons clustered tightly together within the morphospace. However, the ontogeny of enantiornithines is still highly uncertain, so *Chiappeavis* may have developed talons better-adapted to taking prey when fully mature. This can only be tested with the discovery of additional *Chiappeavis* specimens.

### Mechanical advantage

The addition of lower jaw functional indices improves the resolution of the extant MA data, whereas changes in diet categories had little effect (see the supplemental discussion for details).

The jaw mechanics of pengornithids do not point to any particular diet because of MA and functional indices poorly separating diets overall, but some of the more distinct diets can be ruled out. Husking granivores are completely separate from pengornithids in every functional morphospace (Figure 3) so seed cracking can be ruled out with high confidence. Pengornithids also lack the adaptations for increased bite force and bending resistance seen in swallowing granivores and tetrapod hunters, which renders these diets unlikely. Invertivory, piscivory, nectarivory, generalist feeding, and frugivory cannot be ruled out as diets for pengornithids by this line of evidence. *Pengornis* is recovered as most likely to be a generalist feeder, and *Parapengornis* as most likely to be either invertivorous or piscivorous (Table S7). *Yuanchuavis* is predicted as most likely to be a nectarivore (Table S7), driven by its high AMA and PMA combined with low MMH and AMH. The lower jaw of *Yuanchuavis,* then, is adapted to exert relatively high forces during a bite, but not to resist bending forces produced by said bite. The lower jaw of *Yuanchuavis* may gain some selective advantage from flexibility (e.g. lateral^37^ or ventral^38^ bending) of the mandible, such as that which aids insect capture in certain Strisores, including hummingbirds, who must supplement their nectarivorous diet with insect protein.^38^ Many Strisores also have a mandibular curve reminiscent of that seen in *Yuanchuavis* (Figure S1C), which shortens the jaw and gives the group a high jaw-closing mechanical advantage.^23^

**Figure 5:**
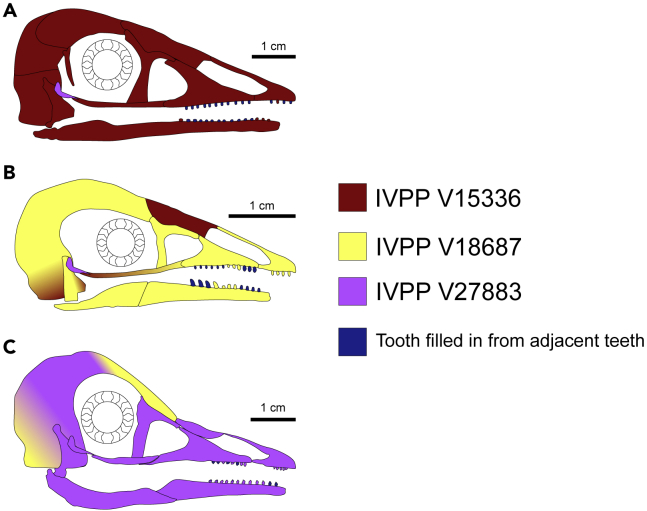
Reconstructions of pengornithid skulls Reconstructions are of *Pengornis* (A), *Parapengornis* (B), and *Yuanchuavis* (C). Colors of different bones indicate which specimen that bone is based on, or that empty tooth sockets were filled with the adjacent tooth. All sclerotic rings are based on *Longipteryx* specimen BMNHC Ph-930B. See the STAR Methods section for more details on reconstruction. Scale bars are based on IVPP V15336 (A), IVPP V18687 (B), and IVPP V27883 (C).

As previously noted,^7^ assignment of fossil taxa as folivores and scavengers is sensitive to upper jaw OMA, which in turn is strongly affected by the position of the quadrate. The quadrate is in place in the holotype of *Pengornis*,^16^ but its position is uncertain in *Parapengornis* and *Yuanchuavis*. A sensitivity analysis moving the quadrate to extreme anterior and posterior positions (Figure S8 and Table S12) found the same result as our previous work^7^: an increase in likelihood of scavenging for posterior shifts of the quadrate, and for folivory increase with anterior shifts of the quadrate (though folivory never became likely for *Yuanchuavis*). Results for *Parapengornis* changed dramatically in the anterior-shifted quadrate model, additionally recovering swallowing granivory as a likely diet. The low, smooth portion of the angular-surangular region in *Parapengornis* extends anteriorly far into the orbit (Figures 5 and S5), meaning that while results from the anteriormost possible point for the quadrate cannot be ruled out, they are not considered biologically likely. Thus, with this line of evidence we cannot rule out scavenging for *Yuanchuavis* or folivory, scavenging, or swallowing granivory for *Parapengornis*.

### Finite element analysis

Changes in diet categories have improved the resolution of extant FEA data (see the supplemental discussion for details).

Pengornithid jaws range from weak to intermediate in strength. *Pengornis* has a weak jaw, with an MWAM strain (275 με) above most herbivores and omnivores (Figure S6), though FDA finds that *Pengornis* has a strong affinity with generalist feeders and folivores (Table S10). *Yuanchuavis* has a somewhat stronger jaw, with an undiagnostic intermediate MWAM strain (199 με) and FDA finds affinity with swallowing granivores and generalist feeders (Table S10). *Parapengornis*’ jaw is slightly stronger than that of *Yuanchuavis* (MWAM 190 με), with FDA finding affinities with swallowing granivores and tetrapod hunters (Table S10). All pengornithids have invertivory and piscivory as likely diets in FDA, and husking granivory and frugivory as unlikely.

### Pengornithid ecology and evolution

Table 2 provides a summary of the palaeodiet interpretations of each line of evidence, and highlights where they agree. The diet of *Pengornis* is the clearest among pengornithids. The genus is considered most likely to have been a generalist feeder, though piscivory is also likely with only subjective elements against it. Generalist feeding is supported by MA and FEA results, with FDA posterior predictions for both MA and FEA finding generalist feeding the most likely diet for *Pengornis*. Generalist body mass is broadly distributed in the dataset, so mass is uninformative of this diagnosis. The unique teeth of *Pengornis* among pengornithids also point to a varied diet. *Pengornis* has two distinct tooth morphotypes: The mesial teeth, which are mostly straight and conical, and the lateral teeth, which are low-crowned and rounded^9^^,^^16^ (Figure 5A). The pattern is similar to the red tegu *Salvator rufescens*, a generalist feeding lizard.^39^ In general, increased heterodonty is associated with an increase in plant intake in squamates.^40^ Talons adapted for raptorially taking large prey are uncommon among extant generalist birds, though caracaras often hunt raptorially and are known to take a large variety of animal prey and occasional plant matter.^41^ Caracaras are unusual among raptors in that they are largely terrestrial,^41^ and as one would expect from this, their claw curvature is relatively low (average 87°), much less than *Pengornis* (average 115°). If caracaras are a valid analogue for *Pengornis*, *Pengornis* was likely more arboreally inclined.

**Table 2:** Summary table of interpretations of each line of evidence used herein

Line of Evidence	Taxon	Likely Diets/Ecologies	Unlikely Diets/Ecologies
Body mass	*Chiappeavis*	Folivory, frugivory, generalist feeding, piscivory, scavenging, tetrapod hunting	Granivory, invertivory, nectarivory
Body mass	*Eopengornis*	Folivory, frugivory, generalist feeding, piscivory, scavenging, tetrapod hunting	Granivory, invertivory, nectarivory
Body mass	*Parapengornis*	Folivory, frugivory, generalist feeding, **piscivory**, scavenging, tetrapod hunting	Granivory, invertivory, nectarivory
Body mass	*Pengornis*	Folivory, frugivory, **generalist feeding**, **piscivory**, scavenging, tetrapod hunting	Granivory, invertivory, nectarivory
Traditional morphometrics	*Chiappeavis*	Non-raptorial perching, ground	Shrike-like
Traditional morphometrics	*Eopengornis*	Shrike-like	Ground
Traditional morphometrics	*Parapengornis*	Small raptor, shrike-like	None
Traditional morphometrics	*Pengornis*	Large raptor	Shrike-like
Mechanical advantage	*Parapengornis*	Generalist feeding, invertivory, nectarivory, **piscivory**	Husking granivory, tetrapod hunting
Mechanical advantage	*Pengornis*	Frugivory, **generalist feeding**, invertivory, **piscivory**	Folivory, husking granivory, tetrapod hunting
Mechanical advantage	*Yuanchuavis*	Frugivory, **invertivory**, nectarivory, **piscivory**	Folivory, husking granivory, tetrapod hunting
Finite element analysis	*Parapengornis*	Swallowing granivory, invertivory, **piscivory**	Folivory, husking granivory, nectarivory, scavenging
Finite element analysis	*Pengornis*	Folivory, **generalist feeding**, invertivory, **piscivory**	Granivory, nectarivory
Finite element analysis	*Yuanchuavis*	Generalist feeding, swallowing granivory, invertivory, piscivory	Husking granivory, scavenging, tetrapod hunting

Body mass, MA, and FEA inform diet. TM informs pedal ecology. See relevant discussion sections for additional details. Bolded diets are agreed upon by all available diet proxies.

Although generalist feeding is considered most likely for *Pengornis*, piscivory and invertivory both merit discussion. Body mass, MA and FEA data all find piscivory as a likely diet in *Pengornis*, and such a diet would be consistent with its raptor-like claws in TM. It has the weakest jaw among pengornithids (Figure S6), typical of extant avian piscivores,^7^ and a low OMA and AO (Figures S3E, S4A and S4B) believed to help piscivorous birds snap up and swallow prey.^7^ Most of *Pengornis*’ teeth, however, are low and globular^9^^,^^16^ (Figure 5A), counter to the narrow conical teeth typical of toothed piscivores. These have been interpreted as “well adapted for crushing relatively hard food items”^20^ pg. 83, which we agree with to some extent but consider true durophagy unlikely given the jaw’s low overall strength (Figure S6). The rostral-most teeth are more conical, and the bluntness of these teeth in the holotype (Figure 5A) may be because of dental wear^16^ (though piscivore teeth in reptiles experience little microwear relative to other diets^42^). Although it is possible fish were caught in the mesial teeth and channeled backwards over the blunt lateral teeth, blunt teeth would reduce grip on a struggling fish and make prey escape more likely, which is not ideal for a bird specializing in taking fish. Thus, specialized piscivory appears less likely than generalist feeding in *Pengornis*. Specialized invertivory also bears addressing briefly. Invertivory, previously suggested in *Pengornis*^18^^,^^19^ pg. 136, is indicated by MA and FEA evidence. However, *Pengornis* is more massive than most extant invertivores. Its talons also indicate adaptations for hunting prey too large to be fully encircled in the talons, and even the giant mayflies of the Jehol Biota can be fully encircled by the toes of the much smaller longipterygids (Figure 9 in^7^). Therefore, invertivory seems less likely than either generalist feeding or specialized piscivory for *Pengornis*.

The diet of *Parapengornis* is the next clearest among pengornithids. Husking granivory and nectarivory can be confidently ruled out, but the quantitative data fail to reject any other diet possibilities. Swallowing granivory, predicted as likely by the FDA of FEA intervals for *Parapengornis* and *Yuanchuavis*, can also be ruled out by assuming that swallowing granivory requires a gastric mill to grind seeds as in extant birds. There is no evidence for gastric mills in birds outside of Ornithuromorpha.^43^ Piscivory is recovered as the most likely diet for *Parapengornis* overall, though the low confidence in piscivory from any single line of evidence leaves this diagnosis tentative. Body mass, MA, and FEA results for *Parapengornis* are all consistent with piscivory, but the most confident FDA assignment to piscivory is MA, at 46% confidence (Table S7). *Parapengornis*’ OMA and AO are near the upper limit for piscivores (Figures S3E, S3F, S4A and S4B) and its jaw strength is greater than the main piscivore cluster in FEA (Figure 4A), again implying low confidence in assigning a piscivorous diet. Notably, though, its teeth are more consistent with taking fish than *Pengornis*. The teeth of *Parapengornis* are generally sharp and conical, as expected of a piscivore, with the lateral-most preserved dentary tooth noticeably recurved (Figure 5B) which would better prevent slippery prey from escaping during swallowing. The claws of *Parapengornis* indicate adaptations for limited handling of prey with the pes. Extant raptors which specialize in capturing fish in their talons tend to have particularly recurved claws (e.g., average 123° for *Pandion haliaetus*) whereas those of *Parapengornis* are relatively straight (average 88°). If *Parapengornis* was a piscivore, its feeding strategy is expected to resemble the wading behavior of the common black hawk *Buteogallus anthracinus* (average claw curvature 84°) which flushes fish in shallow water before quickly flying away with prey in the claws or jaws.^44^ It has been previously noted that the pygostyle of *Parapengornis* indicates an increase in caudal musculature,^15^ which could help maneuver the long tail feathers away from water during aquatic feeding. The hypothesis that *Parapengornis* was adapted for woodpecker- or treecreeper-like climbing^15^ does not conflict with this dietary hypothesis, as *Parapengornis* lacks the hammering adaptations of woodpeckers^45^ and the thin and recurved bill of treecreepers,^36^ so neither would be considered a modern analogue for *Parapengornis* in diet.

*Yuanchuavis*’ diet is poorly resolved. The only known specimen does not preserve a set of pedal unguals (necessary for TM) nor forelimbs (needed to estimate body mass), meaning only MA and FEA results can be applied. Both lines of evidence agree that husking granivory and tetrapod hunting are unlikely in *Yuanchuavis* (Tables S7 and S10), though the apical recurvature of *Yuanchuavis*’ teeth (Figure 5C) makes us hesitant to completely rule out tetrapod hunting. MA and FEA agree that two other forms of carnivory - invertivory and piscivory - are likely diets for *Yuanchuavis*. Like *Parapengornis* neither diet is predicted with high confidence, though unlike *Parapengornis* the taxon does plot among the main cluster of piscivores and invertivores in the FEA function space (Figure S6A) and has the low OMA and OA characteristic of piscivores (Figures S3E, S3F, S4A and S4B). The potential flexibility in the jaw of *Yuanchuavis* offers little clarification, as jaw flexibility is relevant to both piscivorous and insectivorous taxa because of its role in increasing the bird’s gape size during prey capture. Although these factors lead us to believe piscivory or aquatic invertivory are the most likely diets for *Yuanchuavis*, this conclusion should be considered tentative until additional quantitative lines of evidence become available.

The diets of *Eopengornis* and *Chiappeavis* remain entirely unknown. Only body mass and TM data could be taken for each, and body mass data is inconclusive for *Eopengornis* because of the early ontogenetic stage of the holotype.^14^ TM of *Eopengornis*’ claws finds them most similar to shrikes among the extant bird dataset, so if *Eopengornis* was a carnivore it is expected to have some, but limited, ability to manipulate prey with its hindlimbs. The same is true for *Parapengornis*, and the two taxa also share predominantly conical teeth with slight recurvature^14^ (Figure 5B), so it would be unsurprising for them to have a similar dietary niche. Additional specimens of *Eopengornis* which are fully mature and with skulls preserved in lateral view are necessary to test this hypothesis. *Chiappeavis*, on the other hand, has inconclusive TM results which only point to the claws not being used to take prey. This does not rule out carnivory as prey could still be taken with the jaws, meaning only its large body mass gives an indication of diet. Although it is unlikely to be a granivore, nectarivore, or invertivore, it cannot be determined from these data which of the remaining diets were likely.

Dietary proxies, where applicable, point to pengornithids most likely being carnivores adapted for taking vertebrate prey. This was proposed previously based on their unusually large size among enantiornithines.^8^ Piscivory in particular is indicated by low jaw strength and adaptations for a quickly opening jaw that closes in a scissor-like fashion. *Pengornis* shares these characters, but the quantitative analysis results and its blunted lateral teeth indicate a broader diet, possibly using the lateral teeth to more effectively crush plant matter whereas animal prey was taken with the front teeth. Calcium isotope studies^46^^,^^47^ of Jehol taxa including *Pengornis* would be ideal to confirm this hypothesis. Diet trends within Pengornithidae cannot be meaningfully discussed as the phylogeny of the group is inconsistent^13^^,^^14^^,^^15^^,^^17^ and the diet of *Eopengornis*, the oldest member of the clade,^14^ is the most highly uncertain. This work reinforces the necessity for combining multiple lines of evidence when reconstructing diet in deep time, as the additional lines of evidence applicable to *Pengornis* and *Parapengornis* greatly increase the confidence and precision of their dietary assignments.

Fish consumulites are relatively common among birds in the Jehol Biota,^9^ so their absence in pengornithids, some of which appear adapted for piscivory, bears addressing. It is entirely possible that the small sample size of pengornithids simply misses directly preserved evidence of diet, as only ⅔ of published *Yanornis* specimens preserve a fish consumulite^9^ and only four in 230 examined *Anchiornis* specimens preserve any consumulite.^43^ However, taphonomic biases are also likely at play.^8^ Gut retention times tend to decrease with increased flight activity in extant birds.^48^^,^^49^^,^^50^^,^^51^ Enantiornithines in general are reconstructed as more arboreal than contemporary avialans^3^^,^^52^ and Pengornithidae in particular has been reported as particularly arboreally adapted among enantiornithines^15^ with specialized aerodynamic tail fans in the clade^12^ indicating more active flight. Thus, food taken by pengornithids is expected to remain in the body for a shorter time than their more terrestrial contemporaries, lowering the chance of fossilizing while food is still in the body. Even as additional pengornithid fossils are described, a lack of consumulites alone should not be considered a strong counterargument to the hypothesis that pengornithids are adapted for taking fish.

O’Connor^9^ ^pg. 191^ points to the brachydont (low-crowned) teeth of pengornithids indicating hypocarnivory (little intake of vertebrate tissue), which would contradict piscivorous specialization. The link between the two is unclear; the most extensive study on hypocarnivory^53^ found mammal teeth to generally become more rounded and broad as lineages became hypocarnivorous (not unlike the lateral teeth of *Pengornis*, supporting it as a generalist) but does not comment on crown height. O’Connor^9^ does later assert invertivory as a likely diet for enantiornithines as a whole, so hypocarnivory may have been intended in this way. If so, the assumption may have been that vertebrate prey would wear the teeth more than invertebrate prey and the thin enamel in pengornithids could not withstand this increased wear. However, microwear studies in reptiles have found piscivores to experience less dental wear than invertivores^42^ which would also render this argument against pengornithid piscivory weak.

**Figure 6:**
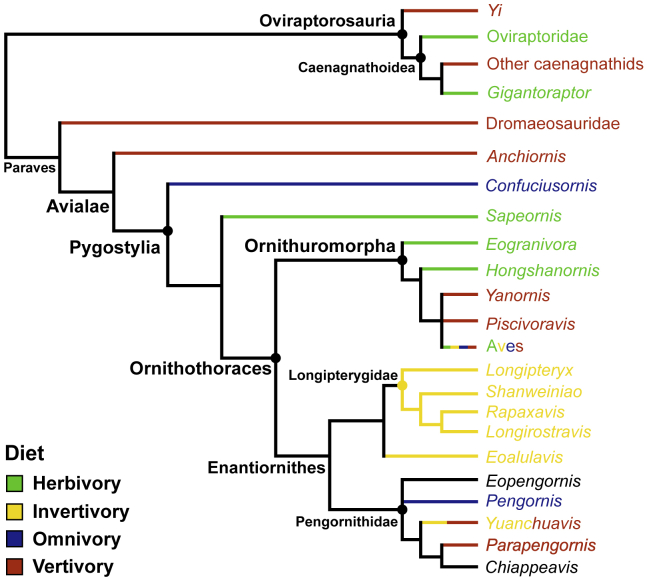
Known pennaraptoran diet, mapped onto a phylogeny The avialan topology is taken from Wang et al.,^17^ with non-avialan branches grafted from Pittman et al.^54^ Oviraptorosaurian,^8^^,^^55^ dromaeosaurid,^56^ and most avialan^7^^,^^8^^,^^52^ diets are mapped based on past works. Note that non-pengornithid avialan diets remain unknown (see Figure 11 in Miller and Pittman^8^ for illustration of this).

Invertivory has been suggested as the ancestral diet for Enantiornithes^9^ ^pg. 191^. Although our findings do not necessarily contradict this, they do highlight the need for further investigation. When plotting diet on a phylogeny (Figure 6), diets are diverse around the Enantiornithes node. We are unable to discern an ancestral diet for Pengornithidae because of uncertainties in the diet of *Eopengornis* and *Chiappeavis*, but from the current data it would likely be either piscivory or macrocarnivorous generalist feeding. Pengornithidae is commonly recovered as one of the earliest-diverging enantiornithine groups with the other known enantiornithine diets of Longipterygidae and *Eoalulavis* deeply nested within Enantiornithes.^8^^,^^17^ It may be, then, that Enantiornithes represents a clade undergoing an initial extreme trophic increase from the ornithothoracine ancestor before subsequent trophic reduction early in the clade’s history.

Alternatively, and more likely in our opinion given the unique morphology of pengornithids among enantiornithines, Pengornithidae may represent a specialized radiation taking advantage of the abundant small vertebrates in the Jehol Biota.^57^ The enantiornithine family Bohaiornithidae from the same formation has been suggested as a similar radiation for taking vertebrate prey,^8^^,^^58^ particularly fish,^58^ and it is unclear to what extent, if any, these groups partitioned the arboreal piscivorous niche. The early diverging ornithuromorph *Yanornis*^59^ and its close relatives^60^ preserve abundant evidence of piscivory as well, though their terrestrial adaptations^3^ may have been adequate to prevent exclusive competition.

Pedal adaptations for *Pengornis*, already a large bird,^16^ to take large prey indicate a more expansive role of birds in Early Cretaceous ecosystems than previously proposed. Birds of the Jehol Biota have been reconstructed as taking plants, insects, and only the smallest fish.^61^ *Pengornis*, however, display adaptations to take larger vertebrates. For reference, the peregrine falcon *Falco peregrinus*, with body mass and claw morphometrics similar to *Pengornis*, regularly takes prey near 300 g and has been recorded taking prey over 3,000 g.^62^ The ecological tendency to capture and kill larger prey – also known as macrocarnivory - has been qualitatively proposed for the Late Cretaceous family Avisauridae.^63^^,^^64^ Thus, this study extends the avialan macrocarnivory record by at least 35 million years into the Early Cretaceous. This is of particular significance as it suggests that the prevailing view of Mesozoic birds occupying low trophic levels (the ‘LTL hypothesis’) needs to shift pending more rigorous investigation. The qualitative evidence which proposed *Pengornis* as invertivorous is similar to that applied to most Mesozoic birds, so other cryptic trophic diversity will likely be revealed with increased quantitative study.

These findings also suggest that the transition of birds away from macrocarnivory during the evolution of flight^10^^,^^11^ (see also Figure 6) was not a universal trend. Some Mesozoic birds appear to perform the roles played by large living raptorial birds, a niche commonly viewed as exclusive to dromaeosaurids during the Mesozoic.^26^^,^^52^ The morphological evolution of birds has long been viewed as not a linear gradation from dinosaur to bird but a mosaic of ancestral and derived traits,^65^ and Pengornithidae serves as evidence that the ecology of early birds was similarly more complicated than we realized.

### Limitations of the study

As in all paleontological studies, the incompleteness of the individual fossils examined and of the fossil record overall limit the scope of our conclusions. Some lines of evidence are unavailable for some taxa, and reconstructions that combine data from individuals within the same genus - and sometimes between genera - were necessary to create functional models. Limitations of reconstruction are compounded by the currently poor understanding of enantiornithine ontogeny. We maintain transparency of how reconstructions were made in Figure 5. The two-dimensional preservation of pengornithid birds in particular limits our functional analyses to two dimensions. This obscures potentially useful information in the mediolateral dimension. Uncertainty in non-fossilizing input parameters (e.g., bone material properties) means that our finite element results may only be used comparatively, as assumptions made for fossil taxa cannot be validated directly. Additional dietary proxies such as isotope geochemistry and dental microwear are lines of evidence that may support or modify our conclusions in future studies.

## STAR★Methods

### Key resources table

**Table:** 

REAGENT or RESOURCE	SOURCE	IDENTIFIER
**Biological samples**		

Holotype specimen of *Chiappeavis magnapremaxillo*	Shandong Tianyu Museum of Nature (STM)	29-1
Holotype specimen of *Eopengornis martini*	STM	24-1
Holotype specimen of *Parapengornis eurycaudatus*	Institute of Vertebrate Paleontology and Paleoanthropology (IVPP)	V18687
Referred specimen of *Parapengornis eurycaudatus*	IVPP	V18632
Holotype specimen of *Pengornis houi*	IVPP	V15336
Holotype specimen of *Yuanchuavis kompsosoura*	IVPP	V27883
Fossil specimen of indefinite pengornithid	IVPP	V18632
Extant bird unguals	Carnegie Museum of Natural History; Florida Museum of Natural History	Various; see raw data files
Extant bird skulls	Skullsite.com^66^	Various; listed alongside species on website

**Deposited data**		

Raw data, code for analyses herein, interactive HTML graphics, and additional results and discussion of extant data	This paper	https://doi.org/10.17632/7m9hfxgygh.1
Percentile bird diet information	Wilman et al.^67^	NA
Extant avian phylogenetic trees	birdtree.org^68^	https://birdtree.org/

**Software and algorithms**		

Hyper-Works 2019 Student Edition	Altair Engineering, Inc.	https://altairuniversity.com/
CorelDraw X8	Corel Corporation	https://www.coreldraw.com/
R programming language	R Core Team^69^	v 4.1.2
R packages: akima, ape, base64enc, car, caret, ggrepel, gtools, htmlwidgets, mda, MASS, OptimalCutpoints, phytools, plotly, RRPP, tidyverse	Akima et al.,^70^ Paradis and Schliep,^71^^,^^72^ Urbanek,^73^ Fox and Weisberg,^74^ Kuhn,^75^ Slowikowski et al.,^76^ Bolker et al.,^77^ Vaidyanathan et al.,^78^ Hastie et al.,^79^ Venables and Ripley,^80^ López-Ratón et al.,^81^ Revell,^82^ Sievert,^83^ Collyer and Adams,^29^ Wickham et al.^84^	v 0.6-2.3, v 5.5, v 0.1-3, v 3.0-12, v 6.0-90, v 0.9.1, v 3.9.2, v 1.5.4, v 0.5-2, v 7.3-54, v 1.1-5, v 0.7-90, v 4.9.4.1, v 1.1.2, v 1.3.1
R functions for phylogenetic flexible discriminant analysis	Schmitz and Motani^32^	https://github.com/lschmitz/phylo.fda

### Resource availability

#### Lead contact

Further information and requests for related data should be directed to and will be fulfilled by the lead contact, Case Vincent Miller (case.miller@connect.hku.hk).

#### Materials availability

Specimens used herein are curated at public institutions (see key resources table). Specimen access is available to all qualified researchers upon request.

### Experimental model and subject details

#### Extant specimen selection

Most extant specimens come from the dataset of.^7^ Twenty additional bird skulls from skullsite.org^66^ were added to the dataset to increase the sample size and phylogenetic breadth of the frugivore, granivore, and nectarivore categories, which were the smallest samples in.^7^ *Harpactes erythrocephalus* (red-headed trogon) and *Podiceps cristatus* (great crested grebe) were also added as members of bird orders that were not represented in.^7^ In total, the mass, MA and functional index, and finite element analysis portions of this study include: nine folivores, 17 frugivores, 17 generalists, eight husking granivores, eight swallowing granivores, 43 invertivores, seven nectarivores, 15 piscivores, eight scavengers, and nine tetrapod hunters. The claw portion of the extant dataset in^7^ remains unchanged.

#### Fossil specimen selection

Published pengornithid specimens were incorporated as scale photos from the literature.^13^^,^^14^^,^^15^^,^^16^^,^^17^^,^^85^ Higher-resolution scale photographs were taken of the pes of the holotype of *Chiappeavis* (STM 24-1) for TM, as those in the original publication proved to be too blurry to take the measurements needed precisely. Skulls of *Eopengornis*^14^ and a well-studied but phylogenetically indeterminate pengornithid^85^ could not be used for skull reconstruction as both are in dorsal or ventral view. The skull of *Chiappeavis* was also not fit for reconstruction due to its early ontogeny, see Pengornithid skull reconstruction for MA and FEA. *Yuanchuavis* only preserves the digit II ungual, so it is not included in the TM dataset. No additional undescribed specimens of any pengornithid taxon could be located for this study.

### Method details

#### Taxonomic reference

We refer to extant taxa based on their genus and species in the Birds of the World database for consistency.^86^ Within data files, taxa are referred to based on the data source (Skullsite Bird Skull Collection^66^ or museum specimen designation). Comments in data files note where these identifications differ from Birds of the World or the bird diet database EltonTraits 1.0.^67^ Designations and relationships of fossil clades are based on.^17^^,^^54^

The pengornithid specimen IVPP V18632 has previously been referred to *Pengornis*,^85^ *Parapengornis*,^15^ and Pengornithidae indet.^13^ The indeterminate Pengornithidae diagnosis is the most recent one, and the one used in this paper.

#### Diet assignment

Extant bird diet was assigned based on the EltonTraits 1.0 database,^67^ a database recording bird diet in intervals of 10%. Cutoffs for assigning a bird to a diet category generally follow,^7^ with the exceptions noted in the supplemental information. Cutoffs are given in Table 1. Our previous work^7^ used two separate extant datasets, the base dataset and one expanded to include “semi-specialists” who were less specialized in a given diet but expanded the phylogenetic breadth of a diet category by including them. In that study both extant datasets gave similar results, so for simplicity all results reported here include semi-specialist birds.

Our past work^7^ separated both frugivores and invertivores by the hardness of the fruits or invertebrates they ate, with the expectation that these different mechanical properties would separate in functional spaces. However, the groups’ separation was poor in that study and additional testing (Figures S9A–S9D) found minimal change to the data when groups were combined. Thus the “hard frugivore” and “soft frugivore” categories of^7^ have been merged here to frugivore, and the “hard invertivore”, “medium invertivore”, and “soft invertivore” categories of^7^ have been merged here to invertivore.

The “soft invertivores” of^7^ were exclusively birds that specialized in filter feeding, aside from the snail kite (*Rostrhamus sociabilis*). A “filter” category was thus tested, with shearwaters and ducks of genus *Anas* added to the dataset after,^87^ to see if the group was ecologically distinct. Filter-feeding birds were not distinct in data visualizations (Figures S9E and S9F) and separating the category did not change the interpretation of the FDA and phyloHSD results, so the category was not used. A past hypothesis that invertivores may separate on lines of hawking or gleaning prey^7^ was also briefly investigated, using information from Birds of the World^86^ and citations therein to split the invertivores. This split also did not appear meaningful when visualising the data (Figure S10), so an undivided invertivore category was retained.

#### Ecological category assignment

Ecological categories of claw use follow,^7^ with modification to raptorial categories based on the discussion therein. Strike and Restraint categories were previously noted to almost completely overlap, and some members of the Suffocate category which took large prey also clustered near them. We hypothesised that, as previously observed for talon shape and mechanical performance,^27^ prey size may have been the controlling factor. Thus the Pierce, Restraint, Strike, and Suffocate categories of^7^ were combined and split again based on if the bird took small or large prey (*sensu*,^25^ respectively prey that can or cannot be fully-encircled within the talons). This was generally judged by species’ entry in the Birds of the World database, with reference to primary literature therein for details of the species consumed. True shrikes (Laniidae) and bushshrikes (Malaconotidae) were separated into a Shrike category, rather than the large raptor category, because they plot far from other large raptors in PCA and separating them increases Fleiss’ kappa^88^ by 0.1–0.15. Helmetshrikes and relatives (Vangidae) are not included in the Shrike category as they are noted to hunt differently from these groups.^89^ The one helmetshrike in this study, *Prionops plumatus*, is classified as a raptor taking large prey due to notes of it taking unspecified reptiles.^90^ Finally, scavenging birds are here classified as non-raptorial perching birds as they were indistinct from perching birds in PCA and phylogenetic HSD.

#### Pengornithid skull reconstruction for MA and FEA

Final pengornithid skull reconstructions are pictured in Figure 5. Pengornithid skulls are generally very well-preserved and complete, so reconstruction required little extrapolation of bone shape from other taxa. Small areas of extrapolation were necessary, though, to create workable biomechanical models.^91^ As in^7^ all inferences were restricted to the family level, though within Pengornithidae relationships are inconsistent^13^^,^^15^^,^^17^ so rationale for inferences are explained below. The holotype specimens of *Pengornis* and *Yuanchuavis* are considered mature (the latter is based on fusion of compound bones of the hindlimb),^16^^,^^17^ while those of *Parapengornis* and *Chiappeavis* are not mature.^13^^,^^15^ By the skeletal fusion stages of Hu and O’Connor,^92^ the holotype specimen of *Chiappeavis* is more mature than either known specimen of *Parapengornis*. In *Chiappeavis* the astragalus and calcaneum are fused,^13^ but they are unfused in every specimen of *Parapengornis*.^14^ *Parapengornis* displays skull bone fusion typical of mature pengornithids^15^ and its skull shape is generally consistent with that of mature pengornithids, so we believe it is reasonable to reconstruct its skull with mature pengornithid skull material. However, the skull of *Chiappeavis* strongly resembles known juvenile enantiornithines^93^ with its characteristically large orbit and shorter rostrum than mature pengornithids. It is unclear if the *Chiappeavis* holotype is in fact less mature than the *Parapengornis* specimens (the former does seem to have less-developed periosteal surfaces throughout the skeleton^13^^,^^15^) or if the skull of *Chiappeavis* is paedomorphic, but in either case we do not consider it appropriate to use other pengornithid skull material to reconstruct the skull of *Chiappeavis*.

Published images^13^^,^^15^^,^^16^^,^^17^ were imported into CorelDraw X8. Skulls of *Pengornis*, *Parapengornis*, *Chiappeavis*, and *Yuanchuavis* are preserved in lateral view. The skulls of *Eopengornis* and indeterminate pengornithid IVPP V18632 are preserved only in ventral view, so reconstruction was not attempted for these taxa. Skulls were then scaled to all have the same length (from tip of the rostrum to rear of the cranium). Once scaled, each distinct bone or set of bones (e.g. premaxilla + nasal with no clear suture preserved) in each skull was outlined and named according to its identification and source specimen. In every specimen most individual bones of the cranium were indistinct, so a general “cranium” outline was made as well. Once complete, new outlines were made by tracing over the composite of bones to make edges and articulations cleaner. Sutures were not intuited in bone sets so as to not overestimate the precision of the reconstruction. Finally, bones and bone sets were colored based on the specimen they came from. Bones or bone sets that are amalgams of multiple specimens were given gradient fills approximating the regions with greatest contribution from a given specimen.

Missing portions of the cranium in *Yuanchuavis* were filled in from *Parapengornis* as they form a clade in the only topology including the former.^17^ The missing nasal and portions of the cranium in *Parapengornis* were filled in from *Pengornis* based on our subjective observation that their skulls were the most similar of the genera studied. The two also form a clade in two studies,^13^^,^^15^ though a third finds them to have diverged early in the family.^17^ The bone labeled the surangular of *Parapengornis* in^15^ is interpreted as the jugal. For all reconstructions, the position of empty alveoli for bones in lateral view were estimated by aligning the opposite jaw, either exposed in dorsal/lateral view or where the alveoli were filled. When in doubt, the teeth positions of *Pengornis* were used to approximate uncertain alveoli due to its excellent preservation of alveoli. As noted in Figure 5 empty alveoli were assumed to have teeth identical to the closest filled alveolus.

*Yuanchuavis* is the only taxon which definitely preserves the quadratojugal bone,^17^ which was then used for the other pengornithid taxa. Its quadratojugal is indistinguishable from the possible quadratojugal in a referred to specimen of *Pengornis*^85^ so conservation of the element’s shape is likely. The sclerotic ring is not well-preserved in any pengornithid (present but eroded in *Chiappeavis*), so these reconstructions use the sclerotic ring of *Longipteryx* specimen BMNHC Ph-930B.^7^ The overall shape of the sclerotic ring is conserved in Aves, though the shape of the scleral ossicles is not expected to differ between families^94^ like Longipterygidae and Pengornithidae. The sclerotic ring appears to fill most of the orbit in *Chiappeavis*, so a similar relative size was used for other pengornithids. Neither the quadratojugal nor the sclerotic ring affect any quantitative calculations in this study.

### Quantification and statistical analysis

#### Phylogenetic tree topologies and time-scaling

Extant avian phylogenetic trees in this study were taken from birdtree.org.^68^ The supertree in^68^ is time-scaled using Bayesian uncorrelated relaxed molecular clock data from 15 genes in 6,663 extant bird species constrained by seven fossil taxa. All fossil species were placed at the age of their oldest discovery with species divergences taking 1,000 years. The Ornithothoraces node was placed at 145 Ma after Bayesian morphological clock analysis of two independent character sets.^95^^,^^96^ This was necessary as the Brownian motion assumptions of pFDA give inaccurate results when tips are extremely close to the root (Lars Schmidt pers. com. 2022), and *Eopengornis* is the oldest pengornithid, enantiornithine, and ornithothoracine known.^14^ All grafted pengornithid branch lengths were scaled linearly so that the total length of the avian portion of the tree was equal to 94 Ma after the estimate of.^97^

#### Body mass

Body mass estimation for the fossil specimens follows the measurements of,^21^ with the revisions to the regression equation noted in Table 1 in^8^:

ENAN:−2.626+1.528HL+0.34bcL+0.828dHW−1.451UL+0.811dUW+0.378TL

See^21^ for diagram of landmarks for measurements.

Prior to the current study, body mass estimates for the holotype of *Pengornis* were made from direct linear measurements^21^ and estimates for *Eopengornis*, *Parapengornis*, and *Chiappeavis* were made from scaled photographs.^8^ A mass estimate for an indeterminate pengornithid specimen^85^ overlooked in^8^ is provided in this study. These calculated masses are provided in Table S1. This method of mass estimation does not allow mass estimation of *Yuanchuavis*due to its missing forelimbs.

Most extant mass data is consistent with,^7^ with masses of newly-added taxa retrieved in the same way. In short, mass data is taken from.^98^ Average masses for the species are used, with male and female mass weighted equally and subspecies or distinct populations weighted by their reported sample sizes. As is standard,^99^ all masses were Log_10_-transformed before comparison. *Dromaius novaehollandiae* is now included in mass analyses as it is no longer an outlier.

#### Traditional morphometrics

Measurements and landmarks for TM measurements of unguals follow the landmarks of^25^ with modifications from^7^ that allow application to a greater range of fossil taxa. The parameters used in the TM analysis are outer arc curvature (in °) for each digit (I, II, III and IV) and outer arc length of digits I, II and IV expressed as a ratio to the outer arc length of digit III. Extant data are unchanged from.^7^ New measurements of pengornithids were taken from scaled photos in CorelDraw X8.

#### Mechanical advantage and functional indices

All measurements for calculating mechanical advantage and functional indices were taken of images in CorelDraw X8 using the “Parallel Dimension” tool. Although images used herein are unscaled, knowing the absolute scale is unnecessary because only ratios are investigated.

The MA and functional index measurements taken for this study combine those of^7^ for the upper jaw and^100^ for the lower jaw.^7^ found that limiting measurements of mechanical advantage and functional index to the upper jaw yielded poor discrimination of diet, so tests in this work include measurements of the lower jaw as well. The landmarks defined in^100^ did not require any modification for these purposes. Fliess’ Kappa,^88^ comparing predicted and true diets for extant taxa, was comparable for lower jaw and upper jaw measurements alone (both 0.40), but combining the two increased Fliess’ Kappa to 0.64.

#### Finite element analysis

##### Model construction

Most FEA model results are carried forward from.^7^ Models for the lower jaws of pengornithids and newly-added extant birds followed the procedures in.^7^ Homogeneous, isotropic material properties for the skull (E = 7000 MPa, ν = 0.35) and rhamphotheca (E = 3000 MPa, ν = 0.35) were used after.^101^ Properties were assigned assuming dorsoventral thickness of 20% rhamphotheca and 80% bone after.^7^^,^^102^ Plane strain assumptions and relative loading for a constant strain state^103^ made results model-size-independent. Loads were applied using the muscle simulation method of,^104^ with orientation based on dissection diagrams^105^^,^^106^^,^^107^^,^^108^^,^^109^^,^^110^^,^^111^^,^^112^^,^^113^^,^^114^^,^^115^^,^^116^^,^^117^^,^^118^^,^^119^^,^^120^^,^^121^ in extant birds and dinosaur muscle reconstruction^122^ in pengornithids. Constraint from translation in all axes was applied at the articular glenoid, and in dorsoventral translation at the rostral tip of the rhamphotheca or first tooth. All models were created and solved within Hyper-Works 2019 Student Edition (*Hyper-Mesh* and *Optistruct*, Altair Engineering, Inc.,USA).

##### Intervals method

We use the intervals method^34^ to compare the outputs of finite element models. The full range of strain for all models is split into a number of equally-sized intervals, and the percent area of each model under each interval of strain is quantified. Convergence testing was used to determine what number of intervals was optimal. Raw intervals data was transformed before multivariate analysis as it is compositional.^123^ Zeroes were imputed using expected value multiplicative lognormal replacement^124^ with the multLN function in R package zCompositions^125^ version 1.3.4. Then, an isometric log ratio transformation^126^ (ilr function in R package compositions^127^ version 2.0-2) was applied to the primary FEA data and a centered log ratio transformation^123^ (clr function in R package compositions^127^ version 2.0-2) was applied to the data used to plot character weights. Imputation is necessary as the logarithm of zero is undefined. Isometric log ratio transformation more completely removes compositional effects from the data,^126^ while a centered log ratio transformation makes it much easier to interpret character weightings.^123^

In finite element models that have not been directly validated with experimental strain data, absolute values of performance should be used for comparative purposes only (and then, only among models built from the same assumptions, such as the ones used in this study).^128^ The MWAM and interval strain values reported here are therefore appropriate for comparing relative performance among the models in this study, but may not be indicative of actual strains in real bone.

#### Statistical analysis

All analyses of the data were performed in R version 4.1.2,^69^ with scripts available from Mendeley Data: https://doi.org/10.17632/7m9hfxgygh.1. This repository also includes interactive HTML-based graphs of all multivariate analyses made using an R package from Plotly,^83^ version 4.9.4.1. Univariate results in this study are compared in violin plots. When comparing subsets of carnivore and herbivore masses, fossil bird masses were compared to cut-off values found with the R package OptimalCutpoints^81^ version 1.1-5 (function optimal.cutpoints, optimised using Youden Index^30^).

We performed two analyses on each multivariate dataset: principal component analysis (PCA; base R function prcomp) and flexible discriminant analysis^129^ (FDA; mda package for R^79^ version 0.5-2 function fda). Both analyses reduce the dimensionality of data to make interpretations easier. PCA maximizes the total variance explained by view axes, while FDA maximizes the between-group variance explained by view axes. All PCAs in this study used the correlation matrix which brings variables into the same scale. All fossil data points were projected independently into multivariate space (i.e., they were not used in calculating the rotation of the data).

When applying this framework to longipterygids,^7^ linear discriminant analysis (LDA) was used as a discriminant analysis, followed by discriminant analysis of principal components to account for the violated assumptions of LDA. FDA accomplishes the same goal as LDA but is non-parametric,^129^ i.e. it has no assumptions to violate. Comparisons of the two found that FDA tended to produce less separation among groups, but it is the more appropriate test for most biological datasets (including these) which are non-normal and non-independent.

Phylogenetic signal is a potential confounding factor in both PCA and FDA. Bird species are not independent data points as each has some level of phylogenetic relationship to every other species. This non-independence may shape the distribution of the dietary proxies. Using the K_mult_ statistic (see below) significant phylogenetic signal was detected in both the TM and MA/functional index datasets, but not in the FEA intervals data. Thus phylogenetic flexible discriminant analysis (pFDA)^32^ was used to account for the phylogenetic signal in the TM and MA/functional index datasets. pFDA incorporates phylogenetic generalized least squares^130^ into FDA^129^ in an attempt to better define groups given the underlying phylogenetic relationships. When applying pFDA to the MA/functional index dataset, it was also found inappropriate for use there: the pFDA functions recovered an optimal λ of 0, which we expect arises because MA and functional indices discriminate diet poorly in extant birds (see p. 2249 in^32^).

Extant groups with more than one member were compared in terms of TM variables (Table S5), MA variables (Table S8), and FEA intervals (Table S11) using the pairwise() function in the RRPP package for R^29^ (version 1.1.2) to test if they were significantly different from one another. 1,000 permutations were used by convention, with sensitivity analyses finding pvalues to converge before this point. Following^7^ we refer to the output of the pairwise() function when comparing means as “phylogenetic HSD”.

Phylogenetic signal was investigated in each dataset using the K_mult_ statistic,^131^ a summary statistic describing the distribution of high-dimensional traits across a given tree. 1,000 permutations were used by convention. A K_mult_ value of 1 indicates trait distribution matches a Brownian motion model, i.e., traits occur as if they changed randomly across the tree with no selection. Values less than 1 indicate taxa are more different from one another than in a Brownian motion model, values greater than 1 indicate taxa are more similar than expected.^31^ The test also provides a pvalue for the presence of significant phylogenetic signal (null hypothesis of no phylogenetic signal). As recommended by Adams and Collyer,^132^ when K_mult_ was less than 1 but statistically significant phylogenetic signal was detected, K values for each individual input variable were also recorded (Tables S6 and S9). The same code was used to calculate K_mult_ and K values (the equivalent for univariate systems like body mass), as Adams^131^ demonstrated that K_mult_ = K for one-dimensional data.

## Data Availability

• Data including supplemental results and discussion of extant data, interactive graphs, and raw data spreadsheets have been deposited at Mendely Data and are publicly available as of the date of publication. DOIs are listed in the key resources table. • All original code has been deposited at Mendely Data and is publicly available as of the date of publication. DOIs are listed in the key resources table. • Any additional information required to reanalyse the data reported in this work is available from the lead contact upon reasonable request.
