# Supplementary material for: Quantitative investigation of pengornithid enantiornithine diet reveals macrocarnivorous ecology evolved in birds by Early Cretaceous
Source: iScience. 2023 Feb 16;26(3):106211. doi: 10.1016/j.isci.2023.106211 (PMC10009206; doi:10.1016/j.isci.2023.106211)
Supplement: Document S1. Figures S1–S10 and Tables S1–S12 [file mmc1.pdf]

iScience, Volume *26*

Supplemental information

Quantitative investigation of pengornithid enantiornithine diet reveals macrocarnivorous ecology evolved in birds by Early Cretaceous

Case Vincent Miller, Michael Pittman, Xiaoli Wang, Xiaoting Zheng, and Jen A. Bright

Supplemental Figures

Figure S1

Violin plots of extant bird mass by diet. Related to Fig. 1. Diets with the same letter are not significantly different in phylogenetic HSD at the p = 0.05 level (Table S2). A, diets lumped into carnivores, herbivores, and omnivores. B, all diets considered in this study. Diet abbreviations: GranivoreH, husking granivore; GranivoreS, swallowing granivore; Tetra Hunt, tetrapod hunter.

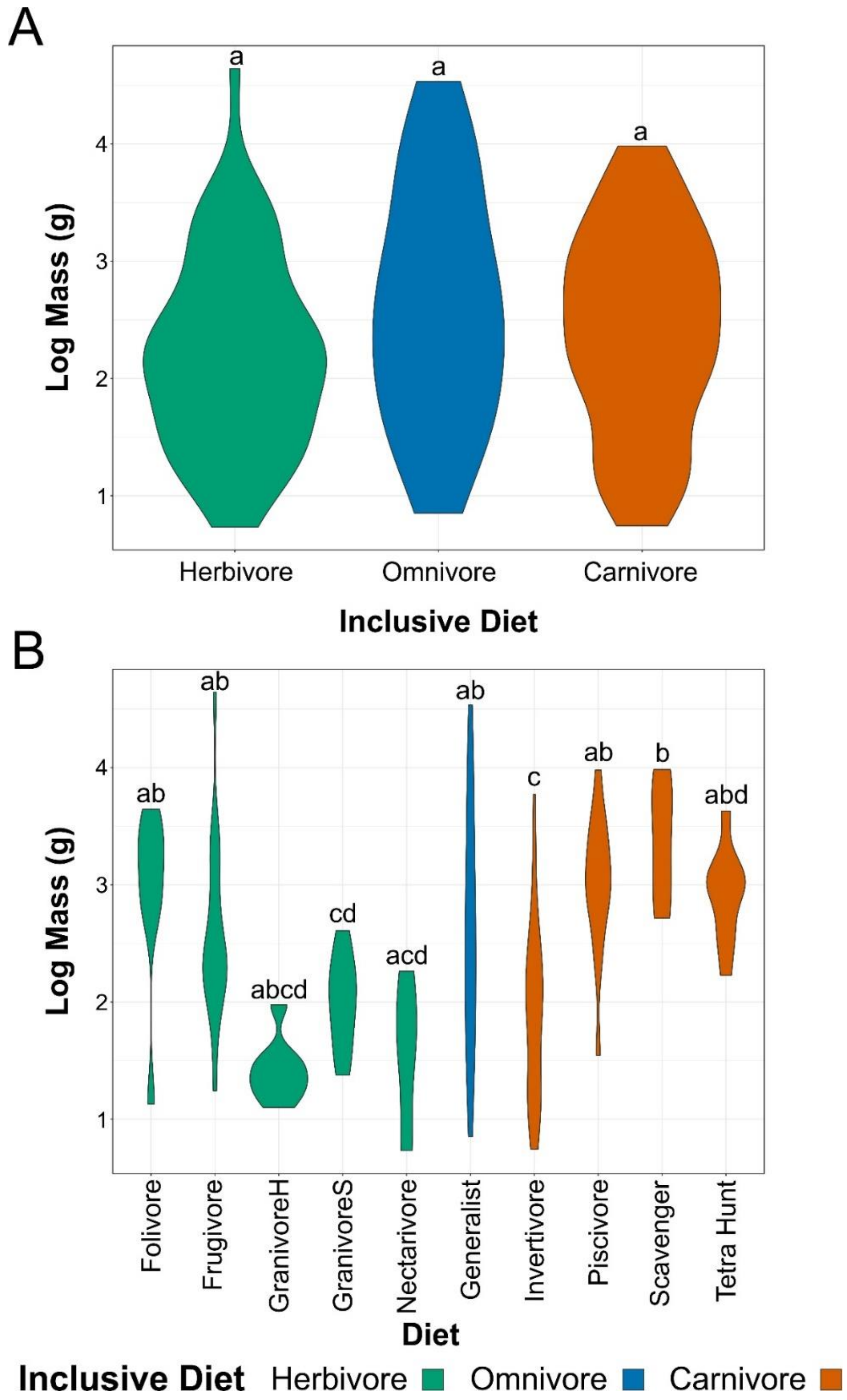

Figure S2

Plot of character weightings for the graphs in Fig. 2. Plots are provided for PCA (A), FDA (B), and pFDA (C).

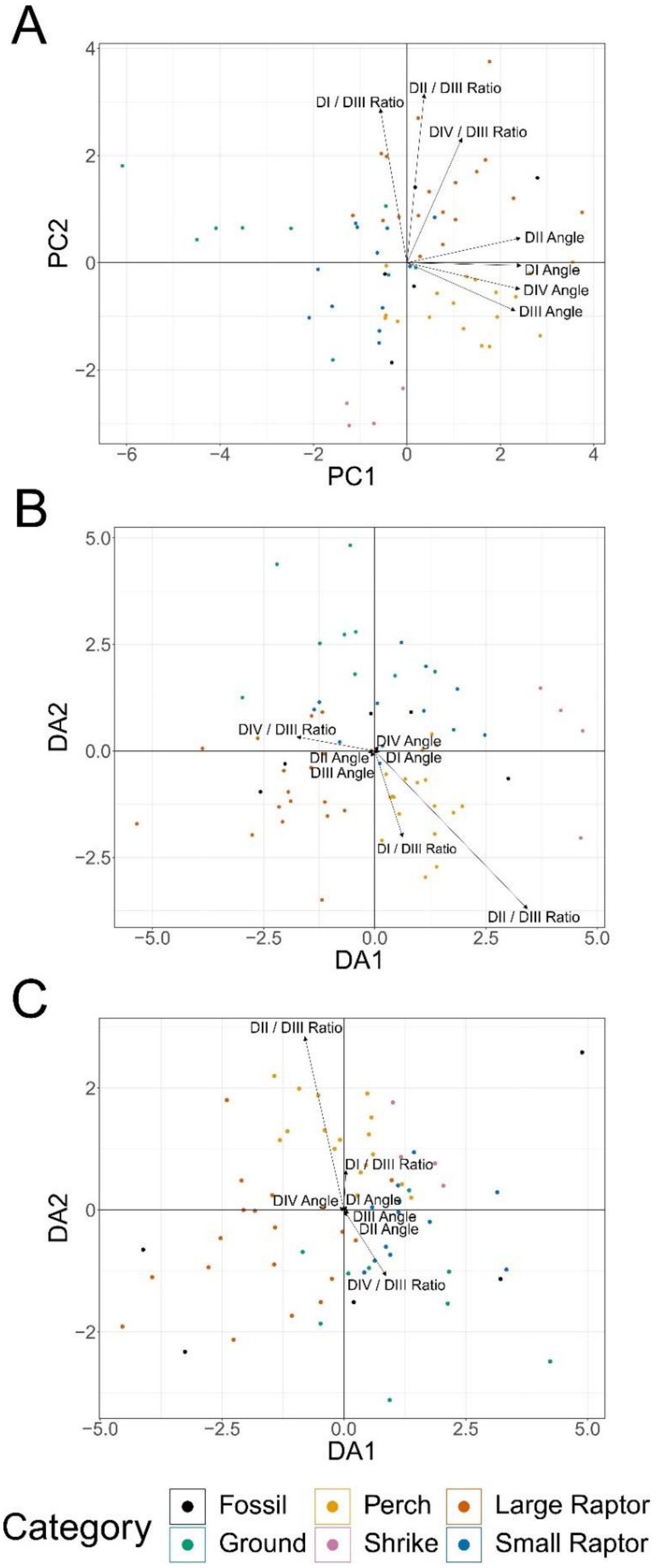

Figure S3

Violin plots of individual functional indices used in this study, part 1 of 2. Related to Fig. 3. Indices include AMA (A-B), PMA (C-D), and OMA (E-F) for the upper jaw (A,C,E) or lower jaw (B,D,F). See Methods section of the main paper for explanation of abbreviations. Diet abbreviations: GranivoreH, husking granivore; GranivoreS, swallowing granivore; Tetra Hunt, tetrapod hunter. Fossil taxon abbreviations: Pa, *Parapengornis*; Pe, *Pengornis*; Y, *Yuanchuavis*.

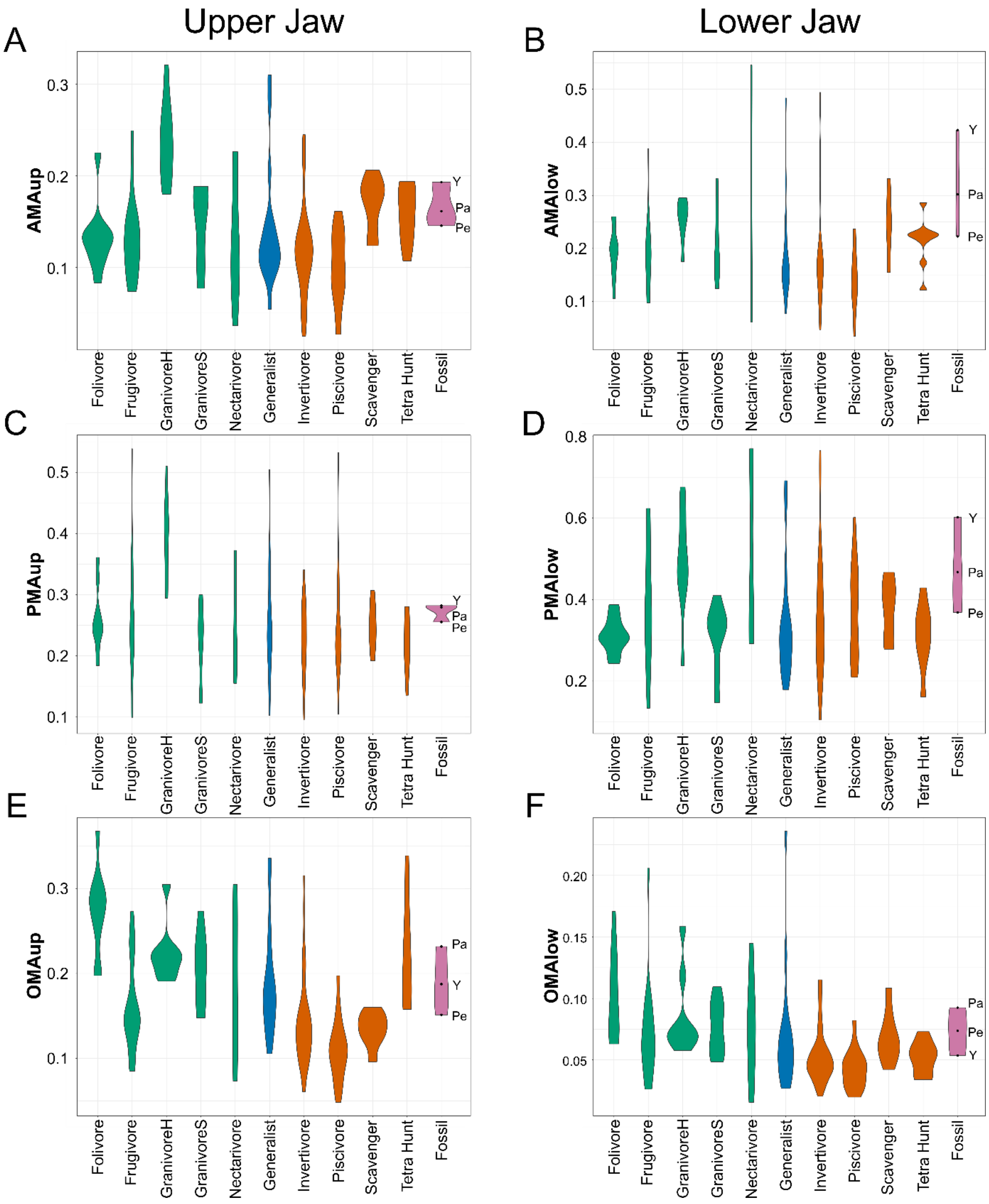

Figure S4

Violin plots of individual functional indices used in this study, part 2 of 2. Related to Fig. 3. Indices include AO (A-B), MCH (C-D), and ACH (E-F) for the upper jaw (A,C,E) or lower jaw (B,D,F). See Methods section of the main paper for explanation of abbreviations. Diet abbreviations: GranivoreH, husking granivore; GranivoreS, swallowing granivore; Tetra Hunt, tetrapod hunter. Fossil taxon abbreviations: Pa, *Parapengornis*; Pe, *Pengornis*; Y, *Yuanchuavis*.

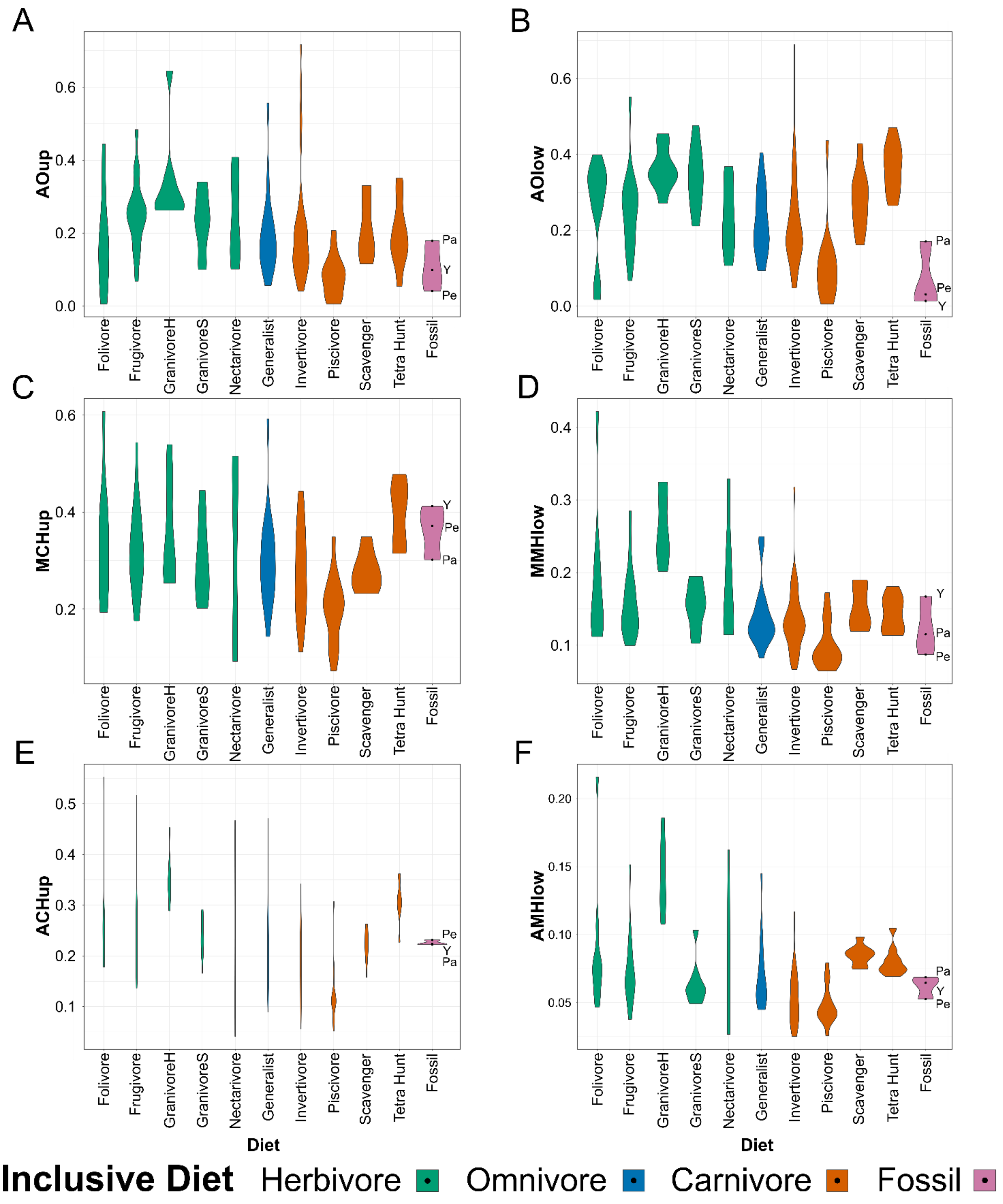

Figure S5

Plot of character weightings for the graphs in Fig. 3. Plots are provided for PCA (A) and FDA (B).

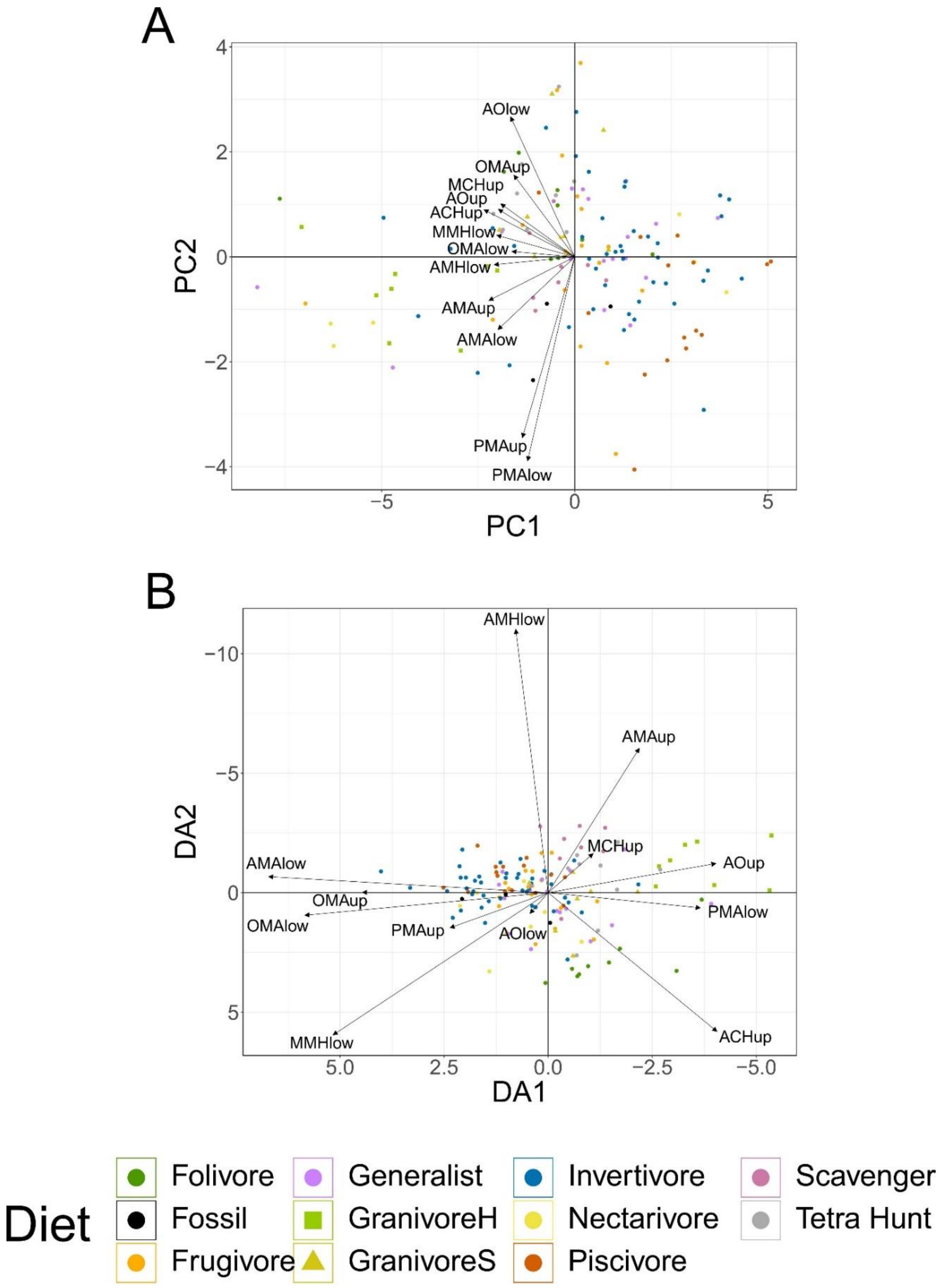

Figure S6

Violin plots of mesh-weighted arithmetic mean (MWAM) strain of extant and fossil bird lower jaw finite element models in this study, organised by more inclusive diets and the whole range of diets considered. Related to Fig. 4. Diets with the same letter above them are not significantly different from one another under phylogenetic HSD of their strain intervals at the p = 0.05 level (Table S11). Diet abbreviations: GranivoreH, husking granivore; GranivoreS, swallowing granivore; Tetra Hunt, tetrapod hunter. Fossil taxon abbreviations: Pa, *Parapengornis*; Pe, *Pengornis*; Y, *Yuanchuavis*.

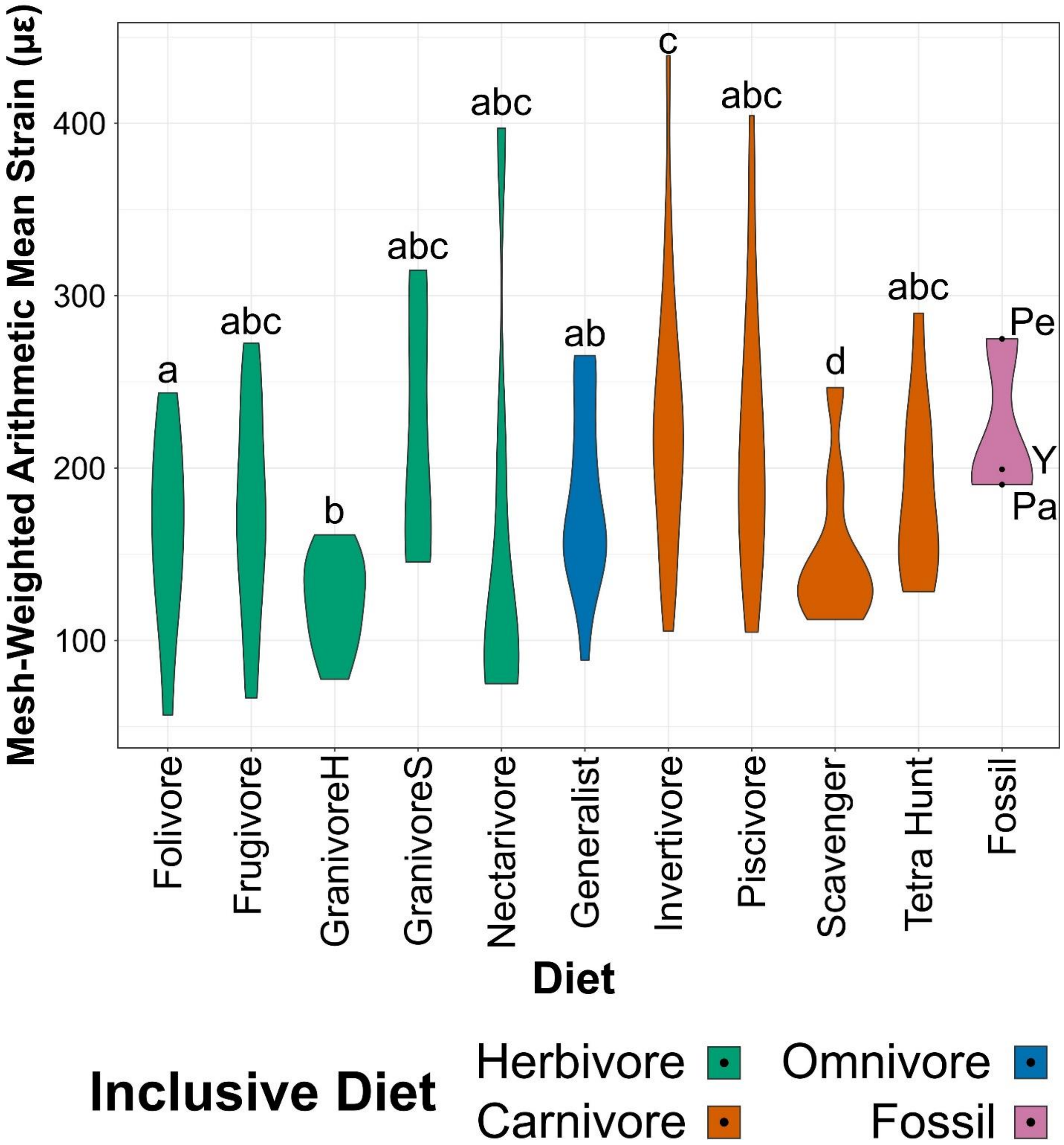

Figure S7

Plot of character weightings for the graphs in Fig. 4. Plots are provided for PCA (A) and FDA (B). V1 is the interval of lowest strain in all graphs. Note that Fig. 4 uses an isometric log-ratio transformation, this plot uses a more easily-interpreted centred log-ratio transformation of the intervals data, so point positions will differ slightly. See the Methods section of the main paper for additional details.

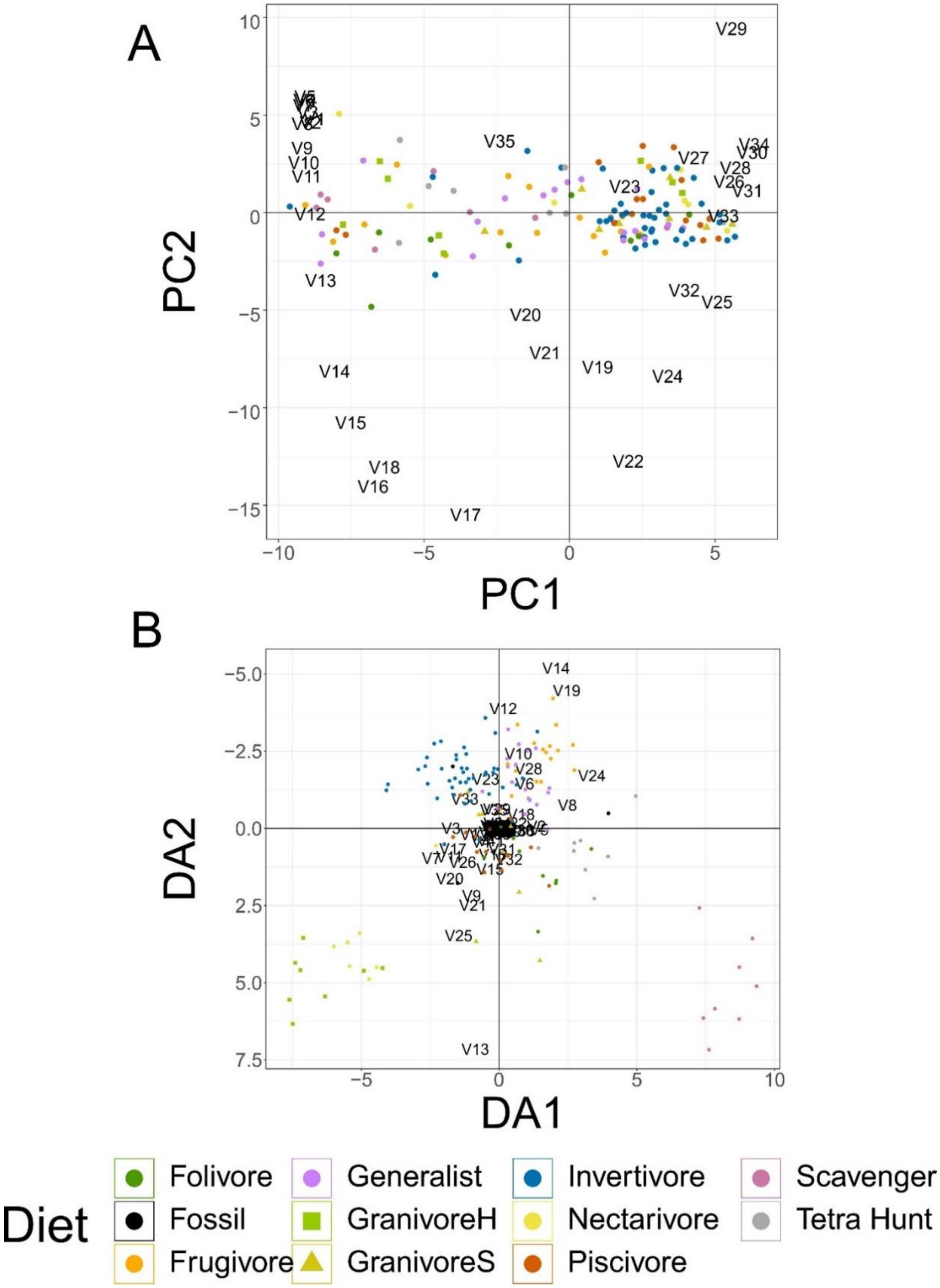

Figure S8

Reconstructions of pengornithid skulls for jaw joint sensitivity analysis. Related to Fig. 3 and Fig. 5. Quadrates are shifted as far anteriorly (A, C) or posteriorly (B, D) as biologically possible to test the effect of their position on MA and functional index results. The quadrate appears *in situ* in IVPP V15336, so the quadrate was not shifted for *Pengornis*. Reconstructions are of *Parapengornis* (A-B) and *Yaunchuavis* (C-D). Colours of different bones indicate which specimen that bone is based on. All sclerotic rings are based on *Longipteryx* specimen BMNHC Ph-930B. See the Methods section and Fig. 5 for more details on reconstruction.

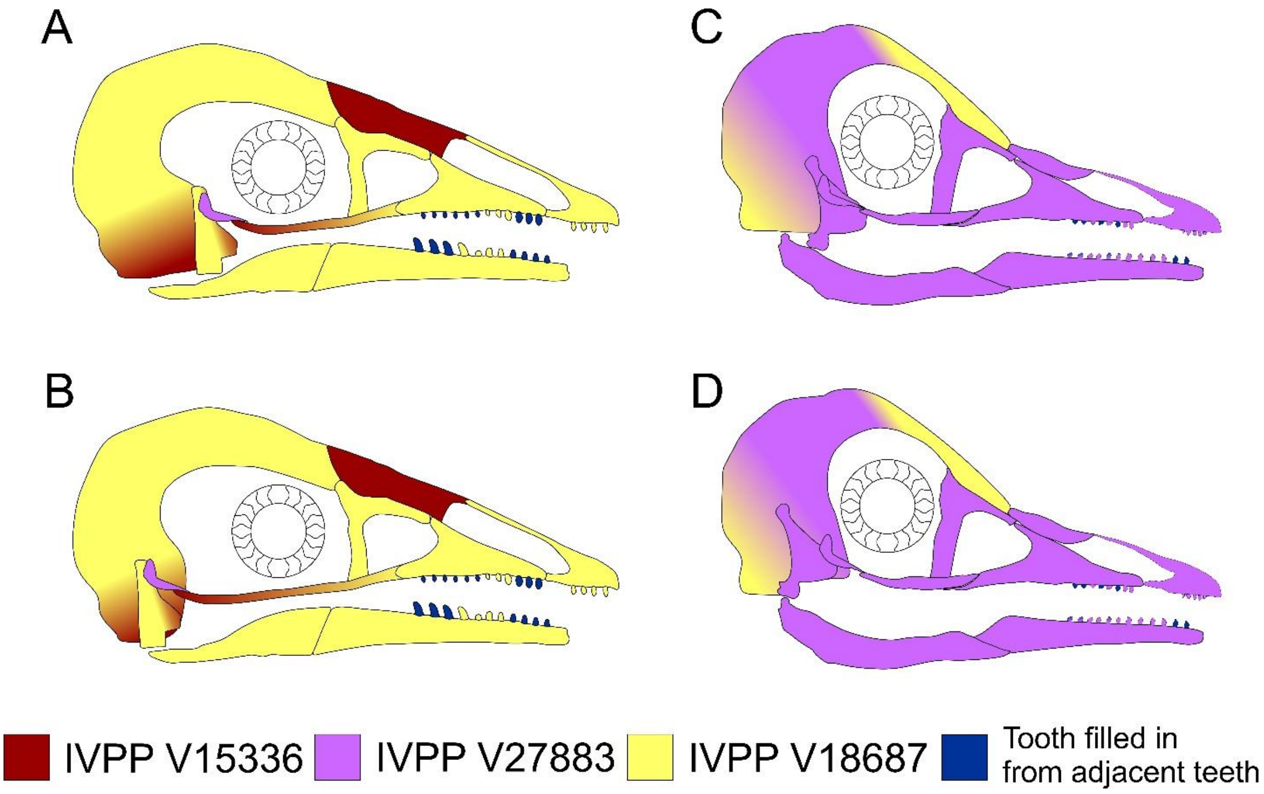

Figure S9

FDA results of MA and FEA intervals data with alternate diet categories, part 1 of 2. Related to Fig. 3 and Fig. 4. Results are shown with invertivores split into hard and medium invertivores (*sensu* [1]; soft invertivores per that study are classified here as medium invertivores as most are filter feeders) (A,B), frugivores split into hard and soft frugivores (*sensu* [1]) (C,D), and filter feeders (*sensu* [2]) separated from other invertivores/folivores (E,F). Graphs represent MA and functional index (A,C,E; compare to Fig. 3B) and FEA intervals (B,D,F; compare to Fig. 4B) data. Note that trends in the data are minimally changed from those shown in Figures 3B and 4B, and that those graphs explain a higher amount of the total variance in the first two discriminant axes. For this reason, we chose not to split the folivore, frugivore, or invertivore categories.

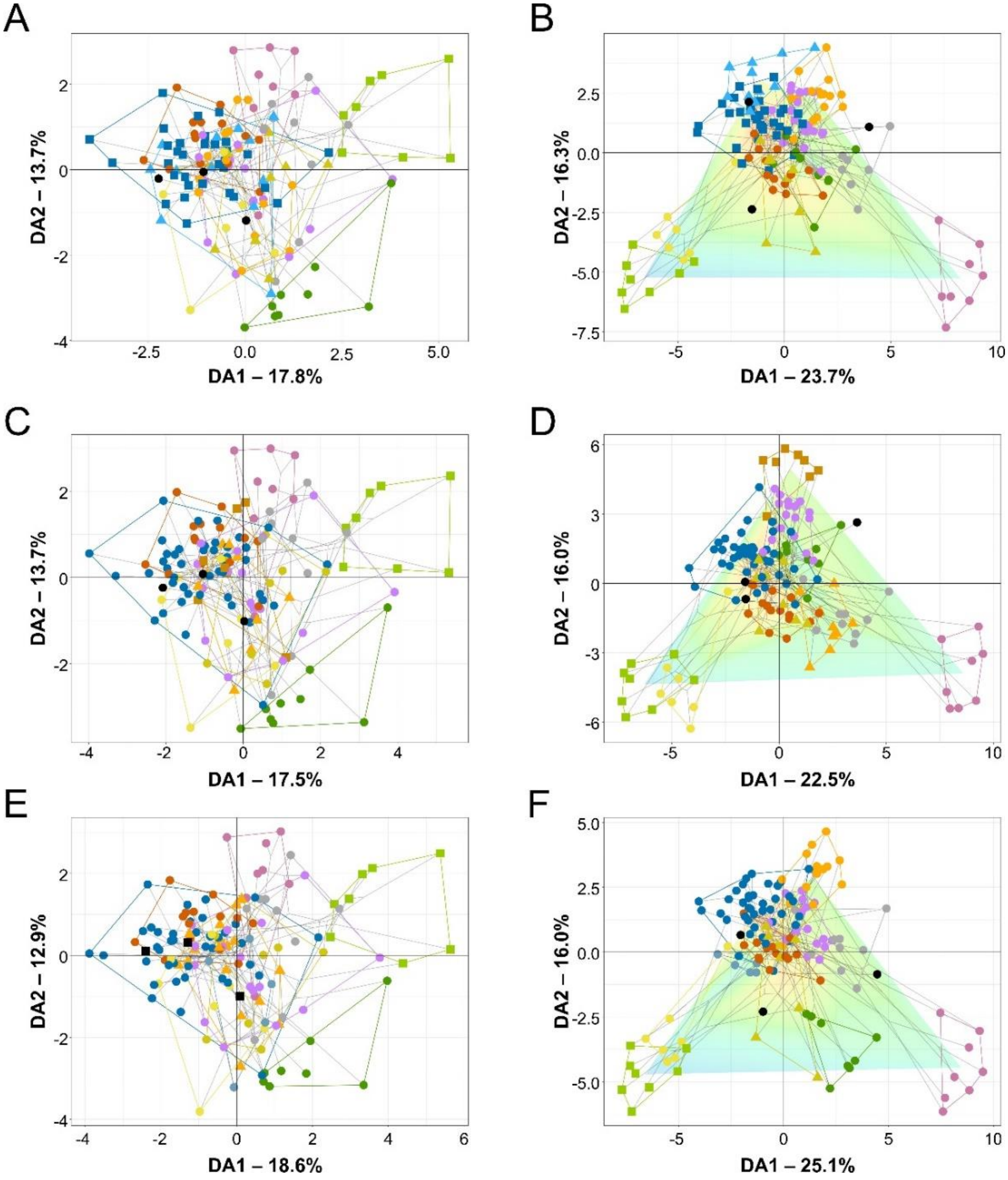

Figure S10

FDA results of MA and FEA intervals data with alternate diet categories, part 2 of 2. Related to Fig. 3 and Fig. 4. Results are shown with invertivores split into gleaning and hawking invertivores (determined by feeding descriptions in [3] and references therein) (A,B). Graphs represent MA and functional index (A; compare to Fig. 3B) and FEA intervals (B; compare to Fig. 4B) data. Note that trends in the data are minimally changed from those shown in Figures 3B and 4B, and that those graphs explain a higher amount of the total variance in the first two discriminant axes. For this reason, we chose not to split the folivore, frugivore, or invertivore categories.

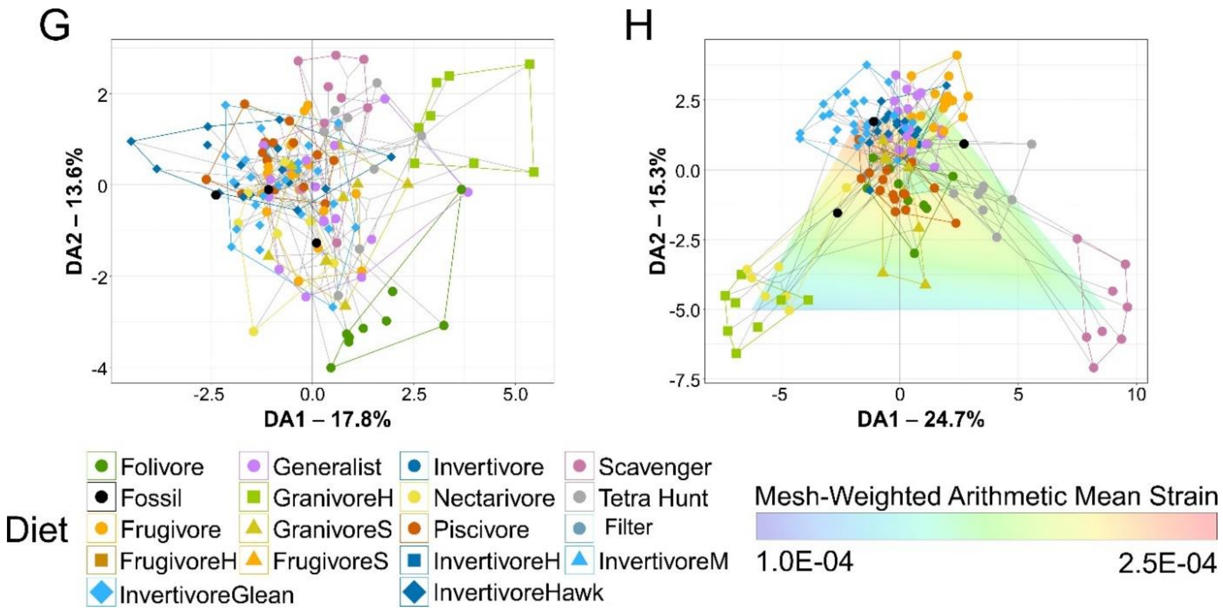

Supplemental Tables

Table S1

Body masses of pengornithids, based on the regression equations of [4]. Related to Fig. 1. Masses are taken from [4,5] and converted into grams for easy comparison to the cut-off points in Fig. 1. A new mass estimate for the indeterminate pengornithid IVPP V18632 is also provided. Note that Table S2 in [5] contains a transcription error for the upper mass estimate of *Parapengornis*, the number here is correct.

Taxon	Specimen Number	Mean Mass Estimate (g)	Lower Mass Estimate (g)	Upper Mass Estimate (g)
Chiappeavis magnapremaxillo (juvenile)	STM 29-11	465	375	556
Eopengornis martini (subadult)	STM 24-1	193	155	230
Parapengornis eurycaudatus	IVPP V18687	429	345	521
Pengornis houi	IVPP V15336	437	352	512
Pengornithidae indet. (subadult)	IVPP V18632	213	172	254

Table S2

p-values for Tukey's HSD and phylogenetic HSD testing if mean mass of birds with different inclusive diets (i.e., combinations of those in Table 2) are different. p-values are indicated with one asterisk (*) for significance at the 0.05 level, two at the 0.01 level, and three at the 0.001 level. Diet abbreviations: FolFrug Folivore + Frugivore; GranNect Granivore + Nectarivore.

Comparison	Tukey's HSD	Phylogenetic HSD
Carnivore-Herbivore	0.473	0.701
Carnivore-Omnivore	0.500	0.744
Herbivore-Omnivore	0.137	0.899
Invertivore-Vertivore	0.000***	0.001***
FolFrug-GranNect	1.90E-06***	0.002**

Table S3

K_{mult}[6] values for all datasets analysed in this paper. Related to Figs. 1–4. Values for body mass are K values but are calculated and interpreted identically. K_{mult} = 1.0 a distribution of measured trait values expected for traits evolving under Brownian motion. Values less than 1 indicate traits more different than expected under Brownian motion and values greater than 1 indicate traits more similar than expected under Brownian motion[7]. p-values are indicated with one asterisk (*) for significance at the 0.05 level, two at the 0.01 level, and three at the 0.001 level. Significant p-values indicate the presence of phylogenetic signal. Note that the K_{mult} function in [6] places a lower limit on the returned p-value, so p-values reported as 1.00E-03 may be more significant.

Dataset	K_{mult}	p-value
All Mass	1.774124	0.001***
Carnivore Mass	1.594164	0.001***
Herbivore Mass	1.949313	0.001***
TM	0.657416	0.001***
MA	0.754337	0.001***
FEA	0.326282	0.411

Table S4

Posterior probabilities predicting pengornithid pedal ecology by FDA and pFDA from TM measurements of bird claws. Related to Fig. 2. Values with green backgrounds are more likely, values with red backgrounds are less likely. Pengornithids generally show affinity with raptorial birds, with less adaptation for prey handling indicated by pFDA than FDA. Diet abbreviations: GranivoreH, husking granivore; GranivoreS, swallowing granivore; Tetra Hunt, tetrapod hunter.

	Taxon	Ground	Perch	Large Raptor	Small Raptor	Shrike
FDA	*Chiappeavis magnapremaxillo*	4.92E-01	1.75E-01	1.10E-01	2.23E-01	1.01E-05
	Eopengornis martini	3.78E-06	9.21E-02	6.25E-05	2.60E-02	8.82E-01
	Parapengornis eurycaudatus	2.35E-02	5.73E-02	1.11E-02	9.08E-01	3.43E-04
	Pengornis houi	3.87E-04	7.68E-03	9.89E-01	2.88E-03	3.05E-13
	Pengornithidae indet.	1.93E-03	4.98E-03	9.75E-01	1.82E-02	8.51E-11
pFDA	*Chiappeavis magnapremaxillo*	0.00E+00	8.04E-01	1.08E-01	8.71E-02	9.46E-04
	Eopengornis martini	0.00E+00	7.80E-05	2.79E-05	3.49E-10	1.00E+00
	Parapengornis eurycaudatus	0.00E+00	1.21E-08	1.38E-06	6.21E-14	1.00E+00
	Pengornis houi	0.00E+00	1.26E-04	2.56E-03	9.97E-01	2.45E-11
	Pengornithidae indet.	0.00E+00	2.80E-03	1.92E-03	9.95E-01	1.74E-09

Table S5

p-values for phylogenetic HSD testing whether unguals of extant birds with different ecological groups have different shape via traditional morphometrics. Related to Fig. 2. p-values are indicated with one asterisk (*) for significance at the 0.05 level, two at the 0.01 level, and three at the 0.001 level. Note that the pairwise() function in RRPP[8] places a lower limit on the returned p-value, so p-values reported as 0.001 may be more significant.

	Ground	Perch	Large Raptor	Small Raptor	Shrike
Ground		0.002**	0.007**	0.054	0.022*
Perch	0.002**		0.905	0.175	0.412
Large Raptor	0.007**	0.905		0.018*	0.244
Small Raptor	0.054	0.175	0.018*		0.397
Shrike	0.022*	0.412	0.244	0.397	

Table S6

K statistics[7] for individual variables used in TM analyses. Related to Fig. 2. K = 1 indicates a similarity of measured traits expected if traits evolved under Brownian motion, values less than 1 indicate traits more different than expected from Brownian motion and values greater than 1 indicate traits more similar than expected from Brownian motion. p-values are indicated with one asterisk (*) for significance at the 0.05 level, two at the 0.01 level, and three at the 0.001 level. Significant p-values indicate the presence of phylogenetic signal. Note that the K_{mult} function in [6] places a lower limit on the returned p-value, so p-values reported as 0.001 may be more significant. These data are unchanged from [1].

	**K**	**p-value**
**DI/DIII Ratio**	0.843968	0.001***
**DII/DIII Ratio**	0.72738	0.001***
**DIV/DIII Ratio**	0.537743	0.001***
**DI Angle**	0.833498	0.001***
**DII Angle**	0.926368	0.001***
**DIII Angle**	0.553193	0.002**
**DIV Angle**	0.424729	0.01**

Table S7

Posterior probabilities predicting pengornithid diet by FDA from MA and functional index data from bird jaws. Related to Fig. 3. Values with green backgrounds are more likely, values with red backgrounds are less likely. Pengornithids generally have high affinity with piscivores and invertivores, with other affinities varying by taxon. Diet abbreviations: GranivoreH, husking granivore; GranivoreS, swallowing granivore; Tetra Hunt, tetrapod hunter.

Taxon	Folivore	Frugivore	Generalist	GranivoreH	GranivoreS	Invertivore	Nectarivore	Piscivore	Scavenger	Tetra Hunt
Parapengornis	2.48E-04	2.57E-02	2.12E-01	8.14E-08	3.85E-03	2.81E-01	1.08E-02	4.62E-01	3.22E-03	1.04E-03
Pengornis	4.05E-02	1.14E-02	7.75E-01	4.20E-07	3.39E-02	5.02E-02	1.56E-02	6.67E-02	6.56E-03	4.28E-04
Yuanchuavis	2.59E-05	2.68E-04	1.82E-02	8.87E-10	2.24E-04	1.59E-01	6.36E-01	1.84E-01	2.74E-03	2.00E-06

Table S8

p-values for phylogenetic HSD testing whether skulls of extant birds with different diets are mechanically different via mechanical advantage and functional indices. Related to Fig. 3. p-values are indicated with one asterisk (*) for significance at the 0.05 level, two at the 0.01 level, and three at the 0.001 level. Note that the pairwise() function in RRPP[8] places a lower limit on the returned p-value, so p-values reported as 0.001 may be more significant. Diet abbreviations: GranivoreH, husking granivore; GranivoreS, swallowing granivore; Tetra Hunt, tetrapod hunter.

	Folivore	Frugivore	Generalist	GranivoreH	GranivoreS	Invertivore	Nectarivore	Piscivore	Scavenger	Tetra Hunt
Folivore		0.153	0.324	0.039*	0.55	0.063	0.236	0.001***	0.035*	0.598
Frugivore	0.153		0.381	0.213	0.86	0.154	0.199	0.001***	0.157	0.368
Generalist	0.324	0.381		0.007**	0.867	0.306	0.31	0.001***	0.007**	0.859
GranivoreH	0.039*	0.213	0.007**		0.134	0.002	0.007	0.001***	0.004**	0.021*
GranivoreS	0.55	0.86	0.867	0.134		0.558	0.381	0.009**	0.229	0.812
Invertivore	0.063	0.154	0.306	0.002**	0.558		0.69	0.001***	0.093	0.841
Nectarivore	0.236	0.199	0.31	0.007**	0.381	0.69		0.435	0.615	0.7
Piscivore	0.001***	0.001***	0.001***	0.001***	0.009	0.001***	0.435		0.226	0.017*
Scavenger	0.035*	0.157	0.007**	0.004**	0.229	0.093	0.615	0.226		0.158
Tetra Hunt	0.598	0.368	0.859	0.021*	0.812	0.841	0.7	0.017*	0.158	

Table S9

K statistics[7] for individual variables used in MA and functional index analyses. Related to Fig. 3. K = 1 indicates a similarity of measured traits expected if traits evolved under Brownian motion, values less than 1 indicate traits more different than expected from Brownian motion and values

greater than 1 indicate traits more similar than expected from Brownian motion. p-values are indicated with one asterisk (*) for significance at the 0.05 level, two at the 0.01 level, and three at the 0.001 level. Significant p-values indicate the presence of phylogenetic signal. Note that the K_{mult} function in [6] places a lower limit on the returned p-value, so p-values reported as 0.001 may be more significant.

Jaw	Measure	K	p
Upper	AMA	0.752924	0.001***
	PMA	0.510357	0.004**
	OMA	0.482913	0.003**
	AO	0.896431	0.001***
	MCH	0.865454	0.001***
	ACH	1.233051	0.001***
Lower	AMA	0.899827	0.001***
	PMA	0.607918	0.001***
	OMA	0.720041	0.001***
	AO	0.849672	0.001***
	MMH	0.713604	0.001***
	AMH	0.821545	0.001***

Table S10

Posterior probabilities predicting pengornithid diet by FDA and pFDA from FEA data from bird lower jaws using the intervals method[9]. Related to Fig. 4. Values with green backgrounds are more likely, values with red backgrounds are less likely. Pengornithids generally have some affinity with piscivores and invertivores, with other affinities varying by taxon. Diet abbreviations: GranivoreH, husking granivore; GranivoreS, swallowing granivore; Tetra Hunt, tetrapod hunter.

Taxon	Folivore	Frugivore	Generalist	GranivoreH	GranivoreS	Invertivore	Nectarivore	Piscivore	Scavenger	Tetra Hunt
Parapengornis	2.97E-23	8.93E-11	1.71E-15	3.17E-23	8.81E-01	1.19E-01	9.56E-23	3.54E-04	3.00E-40	2.24E-06
Pengornis	3.54E-01	4.03E-12	6.46E-01	9.63E-39	2.42E-15	7.71E-08	6.61E-26	4.05E-07	7.53E-15	2.76E-17
Yuanchuavis	6.78E-07	1.80E-08	8.52E-03	1.09E-12	8.89E-01	9.99E-02	5.31E-05	2.67E-03	6.01E-23	9.44E-19

Table S11

p-values for phylogenetic HSD testing whether skulls of extant birds with different diets are mechanically different via comparing strain intervals[9] after finite element analysis. Related to Fig. 4. p-values are indicated with one asterisk (*) for significance at the 0.05 level, two at the 0.01 level,

and three at the 0.001 level. Note that the pairwise() function in RRPP[8] places a lower limit on the returned p-value, so p-values reported as 0.001 may be more significant. Diet abbreviations: GranivoreH, husking granivore; GranivoreS, swallowing granivore; Tetra Hunt, tetrapod hunter.

	Folivore	Frugivore	Generalist	GranivoreH	GranivoreS	Invertivore	Nectarivore	Piscivore	Scavenger	Tetra Hunt
Folivore		0.571	0.331	0.041*	0.055	0.019*	0.351	0.134	0.02*	0.259
Frugivore	0.571		0.996	0.153	0.423	0.149	0.743	0.541	0.004**	0.541
Generalist	0.331	0.996		0.086	0.278	0.023*	0.714	0.258	0.001***	0.316
GranivoreH	0.041*	0.153	0.086		0.06	0.046*	0.374	0.108	0.003**	0.133
GranivoreS	0.055	0.423	0.278	0.06		0.677	0.722	0.569	0.004**	0.63
Invertivore	0.019*	0.149	0.023*	0.046*	0.677		0.867	0.917	0.001***	0.379
Nectarivore	0.351	0.743	0.714	0.374	0.722	0.867		0.823	0.017*	0.734
Piscivore	0.134	0.541	0.258	0.108	0.569	0.917	0.823		0.01*	0.718
Scavenger	0.02*	0.004**	0.001***	0.003**	0.004**	0.001***	0.017*	0.01*		0.003**
Tetra Hunt	0.259	0.541	0.316	0.133	0.63	0.379	0.734	0.718	0.003**	

Table S12

Sensitivity analysis of the position of the quadrate on predicting pengornithid diet (See reconstructions in Fig. S8). Related to Fig. 3. Posterior probabilities are provided from FDA on MA and functional index data from bird jaws. Values with green backgrounds are more likely, values with red backgrounds are less likely. Compared to results with most likely reconstructions (Table S7), folivory is more likely when the quadrate is shifted anteriorly and scavenging and piscivory are more likely when the quadrate is shifted posteriorly. Diet abbreviations: GranivoreH, husking granivore; GranivoreS, swallowing granivore; Tetra Hunt, tetrapod hunter.

	Taxon	Folivore	Frugivore	Generalist	GranivoreH	GranivoreS	Invertivore	Nectarivore	Piscivore	Scavenger	Tetra Hunt
Anterior-Shift	*Parapengornis*	9.95E-01	4.23E-07	4.24E-03	1.46E-09	9.68E-04	3.06E-07	4.09E-08	4.85E-07	1.81E-07	9.10E-08
	Yuanchuavis	5.27E-03	3.52E-04	6.85E-02	3.09E-10	2.41E-03	1.03E-01	7.45E-01	7.40E-02	1.02E-03	1.29E-06
Posterior-Shift	*Parapengornis*	5.66E-07	8.62E-03	6.61E-02	7.51E-06	6.55E-04	1.84E-01	8.32E-03	5.34E-01	1.96E-01	2.21E-03
	Yuanchuavis	1.26E-07	5.49E-05	1.25E-03	5.34E-11	2.21E-05	1.16E-01	7.33E-01	1.44E-01	6.25E-03	8.26E-07

Supplemental References

1. Miller, C.V., Pittman, M., Wang, X., Zheng, X., and Bright, J.A. (2022). Diet of Mesozoic toothed birds (Longipterygidae) inferred from quantitative analysis of extant avian diet proxies. BMC Biol *20*, 101. 10.1186/s12915-022-01294-3.
2. Zweers, G., Dejong, F., Berkhoudt, H., and Vandenberge, J.C. (1995). Filter-feeding in flamingos (*Phoenicopterus ruber*). The Condor: Ornithological Applications *97*, 297-324. 10.2307/1369017.
3. Billerman, S.M., Keeney, B.K., Rodewald, P.G., and Schulenberg, T.S. (2021). Birds of the World. https://birdsoftheworld.org/bow/home.
4. Serrano, F.J., Palmqvist, P., and Sanz, J.L. (2015). Multivariate analysis of neognath skeletal measurements: implications for body mass estimation in Mesozoic birds. Zool J Linn Soc *173*, 929-955. 10.1111/zoj.12215.
5. Miller, C.V., and Pittman, M. (2021). The diet of early birds based on modern and fossil evidence and a new framework for its reconstruction. Biol Rev *96*, 2058-2112. 10.1111/brv.12743.
6. Adams, D.C. (2014). A generalized K statistic for estimating phylogenetic signal from shape and other high-dimensional multivariate data. Syst Biol *63*, 685-697. 10.1093/sysbio/syu030.
7. Blomberg, S.P., Garland, T., and Ives, A.R. (2003). Testing for phylogenetic signal in comparative data: behavioral traits are more labile. Evolution *57*, 717-745. 10.1111/j.0014-3820.2003.tb00285.x.
8. Collyer, M.L., and Adams, D.C. (2018). RRPP: An r package for fitting linear models to high-dimensional data using residual randomization. Methods Ecol. Evol. *9*, 1772-1779.
9. Marcé-Nogué, J., De Esteban-Trivigno, S., Püschel, T.A., and Fortuny, J. (2017). The intervals method: a new approach to analyse finite element outputs using multivariate statistics. PeerJ *5*, e3793.
